# Doping strain induced bi-Ti^3+^ pairs for efficient N_2_ activation and electrocatalytic fixation

**DOI:** 10.1038/s41467-019-10888-5

**Published:** 2019-06-28

**Authors:** Na Cao, Zheng Chen, Ketao Zang, Jie Xu, Jun Zhong, Jun Luo, Xin Xu, Gengfeng Zheng

**Affiliations:** 1 Department of Chemistry, Laboratory of Advanced Materials, Shanghai Key Laboratory of Molecular Catalysis and Innovative Materials, Collaborative Innovation Center of Chemistry for Energy Materials, Fudan University, Shanghai, 200438 China; 2 Center for Electron Microscopy and Tianjin Key Lab of Advanced Functional Porous Materials, Institute for New Energy Materials, School of Materials, Tianjin University of Technology, Tianjin, 300384 China; 3 Jiangsu Key Laboratory for Carbon-based Functional Materials and Devices, Institute of Functional Nano and Soft Materials (FUNSOM), Soochow University, Suzhou, 215123 China

**Keywords:** Catalyst synthesis, Electrocatalysis, Nanoscale materials

## Abstract

The electrochemical N_2_ fixation to produce ammonia is attractive but significantly challenging with low yield and poor selectivity. Herein, we first used density function theory calculations to reveal adjacent bi-Ti^3+^ pairs formed on anatase TiO_2_ as the most active electrocatalytic centers for efficient N_2_ lying-down chemisorption and activation. Then, by doping of anatase TiO_2_ with Zr^4+^ that has similar *d*-electron configuration and oxide structure but relatively larger ionic size, the adjacent bi-Ti^3+^ sites were induced and enriched via a strained effect, which in turn enhanced the formation of oxygen vacancies. The Zr^4+^-doped anatase TiO_2_ exhibited excellent electrocatalytic N_2_ fixation performances, with an ammonia production rate (8.90 µg·h^−1^·cm^−2^) and a Faradaic efficiency of 17.3% at −0.45 V versus reversible hydrogen electrode under ambient aqueous conditions. Moreover, our work suggests a viewpoint to understand and apply the same-valance dopants in heterogeneous catalysis, which is generally useful but still poorly understood.

## Abstract (web summary)

Electrocatalytic N_2_ conversion to ammonia offers a low-carbon alternative to industrial ammonia production, although the rational design of active, efficient catalysts remains limited. Here, authors show Ti^3+^-pair sites in anatase TiO_2_ to be active for aqueous N_2_ electroreduction to ammonia.

## Introduction

The production of ammonia (NH_3_) by the well-known Haber‒Bosch process from N_2_ and H_2_ has marked over a century of success for providing > 80% nitrogen source for fertilizer and an alternative energy carrier with large energy density^1^. Despite the natural abundance of N_2_, the high bond energy of the N≡N triple bond (941 kJ mol^−1^) prevents it as a reactive form and thus demands a significant amount of the global energy cost annually^2^. In addition, the use of fossil fuels to produce H_2_ reactant also leads to a significant level of CO_2_ release^3^. The electrochemical N_2_ fixation (also known as N_2_ reduction reaction, N_2_RR) can be processed in ambient conditions and use inexpensive aqueous electrolytes as the proton source, and thus is regarded as a promising alternative approach^4^. The direct electron transfer from electrode surface to N ≡ N requires overcoming substantially high energy barriers^5^, so the key to achieve efficient N_2_ fixation is to develop active catalytic centers that can efficiently reduce the large activation barrier of N ≡ N and promote its dissociation. As an overwhelming level of water molecules exists than solvated N_2_, the other key to achieve this goal is to enrich the electrocatalytic centers that can favorably proceed with the N_2_RR over the hydrogen evolution reaction (HER).

Theoretical calculations have suggested that metal sites with low chemical valence are potential to enhance the electron-donating ability to the π* antibonding orbitals of N_2_ molecule^6^, which weakens the N≡N bond and drives the eventual cleavage of N_2_. Some noble metal-based catalysts such as Ru^7^ and Au^8^ with stepped surfaces can strongly bind to N_2_ and the intermediates, and thus can lower the overpotential and increase the rate of the N_2_RR in an aqueous electrolyte. Earth-abundant compounds^4,9^, including metal oxides, nitrides and carbides, have also been investigated as electrocatalysts^10^, with tailorable activities by specific facets^11^, defects^12^, vacancies^13^, or hybrid material interfaces^14^. Nonetheless, to date, the electrocatalytic N_2_ fixation is still limited by its low yields and slow kinetics^15^. The critical understanding and rational tuning the active centers of N_2_RR electrocatalysts remain as a highly challenging but imperative issue.

Previously, the roles of oxygen vacancies (Vo’s) in transition metal oxides have been extensively discussed^16–18^, and the low-valance dopants have also been suggested to facilitate the formation of Vo’s^19^, but the knowledge of their contributions to the N_2_RR is still limited. For example, Li et al. reported that TiO_2_ with Vo’s can chemisorb and activate N_2_ molecules^16^, and the formation of each Vo’s is, in turn, related to the formation of a pair of Ti^3+^. Hirakawa et al.^17^ suggested that two Ti^3+^ ions in adjacent positions, which are inherently created on the surface defects of rutile TiO_2_ (110) surfaces, behave as active sites for photocatalytic conversion of N_2_ to ammonia with water. However, more-recent calculations provide different results and indicate that such rutile TiO_2_ (110) surfaces are unlikely to be the relevant surface for the N_2_RR^20^. To date, all the theoretical and experimental studies have not been able to reconcile the discrepancies in which types of TiO_2_ are the best for the N_2_RR, or what is the bonding nature of the active centers.

Herein, by means of density function theory (DFT) calculations, we first reveal that two adjacent Ti^3+^ sites (designated as a bi-Ti^3+^ pair) on anatase TiO_2_ (101) can chemically adsorb and activate N_2_ molecules in a lying-down manner, whereas single or isolated Ti^3+^ sites cannot. On the other hand, similar bi-Ti^3+^ pair on rutile (110) surface cannot adsorb and activate N_2_, unless the strong repulsion between one of its nearest lattice oxygens at the bridge sites and the negatively charged N_2_ upon activation can be eliminated. Thus, the formation of two adjacent Ti^3+^ sites on anatase (101) should be the most-effective electrocatalytic centers for N_2_ fixation.

**Fig. 1:**
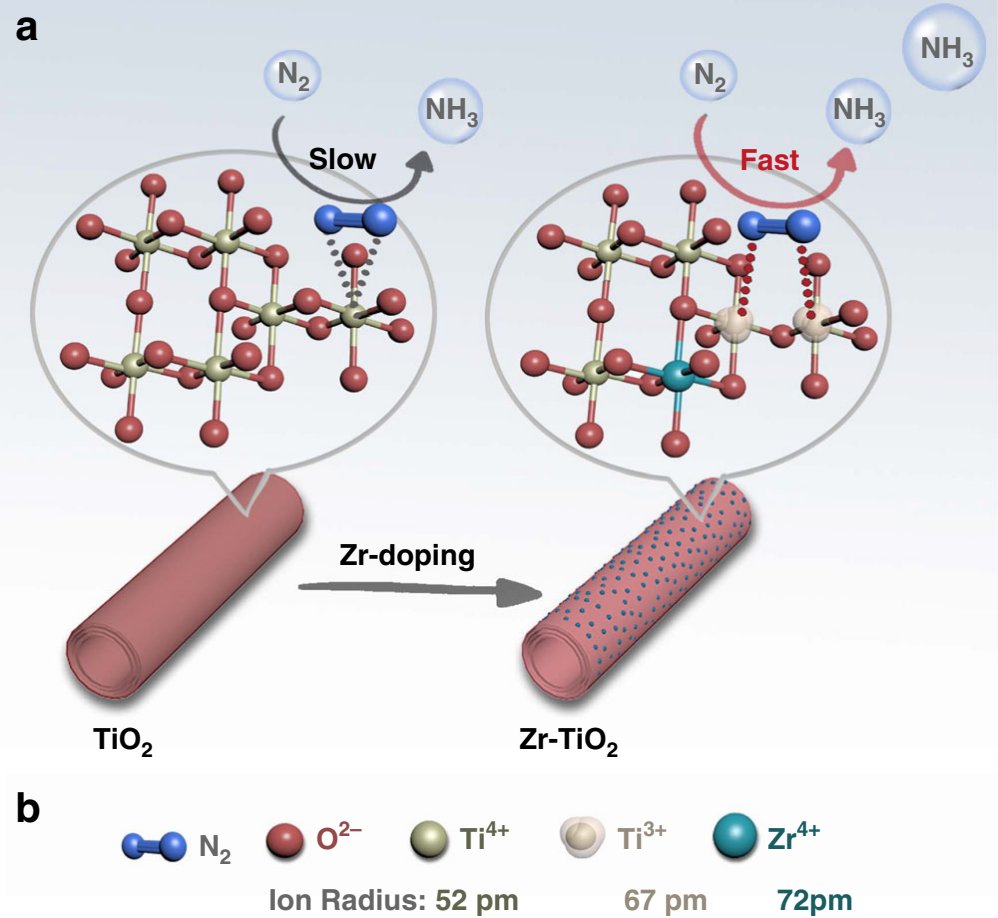
Schematic illustration of N_2_ fixation and activation. **a** The formation of an oxygen vacancy (Vo) and adjacent bi-Ti^3+^ sites owing to the Zr-doping in anatase TiO_2_. **b** Comparison of the ionic radius of Ti^4+^, Ti^3+^, and Zr^4+^

Accordingly, we develop an experimental strategy for inducing such adjacent bi-Ti^3+^ sites on anatase (101) surfaces as N_2_RR active centers, using a dopant-induced Vo formation strategy. Zr^4+^ is selected to dope in the TiO_2_ framework, owing to its similar *d*-electron configuration and oxide structure, as well as its suitable size. As shown in Fig. 1, doping of Zr^4+^, which has a relatively larger radius of 72 pm (compared with 52 pm of Ti^4+^)^21^ into anatase TiO_2_, can still retain its anatase crystal structure but also exert a tension on the TiO_2_ framework, which can enhance the formation of Vo. As the oxidation number of Zr^4+^ is fixed, the newly formed Vo must be associated with the formation of two adjacent Ti^3+^ sites, which are beneficial to enrich active centers and enhance the N_2_RR over the HER. In contrast, Ce^4+^ with a much larger ion radius (106 pm) cannot be incorporated into the TiO_2_ framework without breaking the original crystal structure, whereas Ce^3+^ can also be associated with the newly formed Vo’s, thus not contributing to the formation of the bi-Ti^3+^ pairs as active centers. Electrochemical measurements demonstrate that the Zr^4+^-doped anatase TiO_2_ exhibit significant enhanced N_2_RR performances, including an ammonia formation rate of 8.90 ± 0.17 µg h^−1^ cm^−2^ catalyst and a corresponding Faradaic efficiency (FE_NH3_) of 17.3%, significantly surpassing those of undoped TiO_2_ or Ce^4+^-doped TiO_2_, whose efficiencies were limited by their lower Ti^3+^ densities.

## Results and discussion

### Theoretical calculations

In order to tune Ti^3+^-based electrocatalysts for the N_2_RR, DFT calculations were first carried out to rationally screen different types of Ti^3+^ for a better understanding of bonding structures of these active centers. The calculations for screening an ideal N_2_RR electrocatalyst are often based on the following criteria:^22–24^ first, strong chemisorption of N_2_ molecules; second, effective stabilization of N_2_H*; and third, destabilization of NH_2_*. In the present study, we also investigated the capability of forming Vo’s efficiently, as the fourth criterion.

**Fig. 2:**
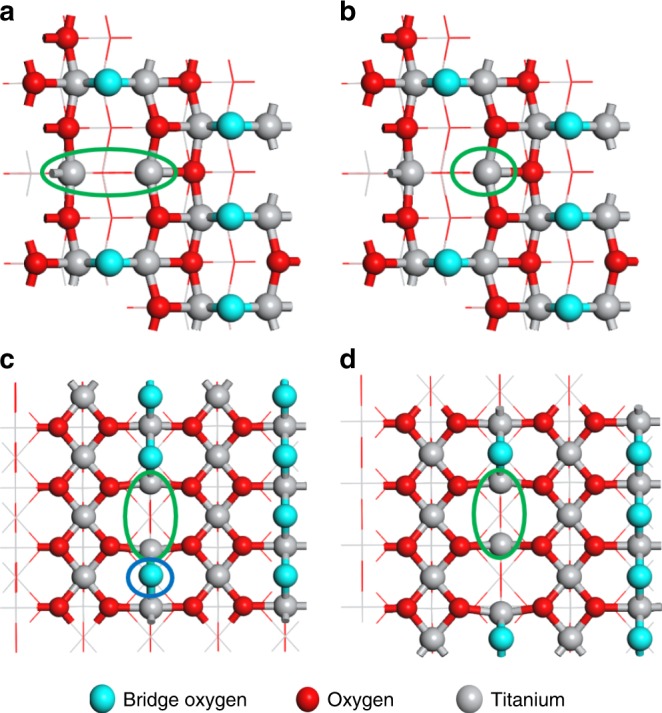
DFT calculations of four types of Ti^3+^ sites. **a** The adjacent bi-Ti^3+^ on anatase (101) surfaces with one oxygen vacancy (i.e., A(101)-Vo); **b** the single Ti^3+^ with four coordination on anatase (101) surfaces with one oxygen vacancy; **c** the adjacent bi-Ti^3+^ on rutile (110) surfaces with one oxygen vacancy (i.e., R(110)-Vo); **d** the adjacent bi-Ti^3+^ on rutile (110) surfaces with a pair of oxygen vacancies together, (i.e., R(110)-2Vo’s). The light-blue spheres stand for the lattice oxygens at the bridge sites where the surface oxygen vacancies are formed most easily. The red spheres stand for the other lattice oxygens on the surfaces and the gray spheres stand for the titanium cations. All four types of the active site models are highlighted by using the green circles. As compared **c** and **d**, the bridge lattice oxygen (circled in blue) can exert a large repulsion with the chemisorbed N_2_ if it adopts a lying-down mode

**Fig. 3:**
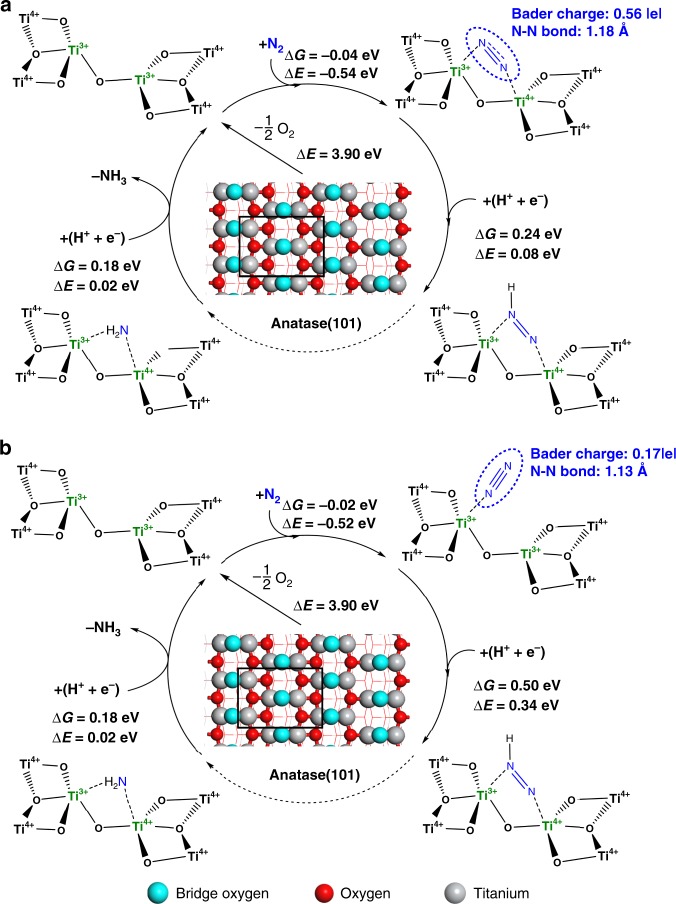
DFT predicted activity for different types of Ti^3+^ site. **a** adjacent bi-Ti^3+^ on anatase (101) surfaces with oxygen vacancy, **b** single Ti^3+^ on anatase (101) surfaces with oxygen vacancy. The light-blue spheres stand for the lattice oxygens at the bridge sites where the surface oxygen vacancies are formed most easily. The red spheres stand for the other lattice oxygens on the surfaces and the gray spheres stand for the titanium cations. Δ*G* refers to the free energy, and Δ*E* refers to the electronic energy. The Vo formation energy, Δ*E*(Vo), was calculated in related to the 1/2 O_2_ formation as described in the Methods section

According to the four criteria above, several types of Ti^3+^ sites were screened for the N_2_RR, including adjacent bi-Ti^3+^ pairs on anatase (101) surfaces (designated as A(101)-Vo), rutile (110) surfaces with 1 or 2Vo’s (designated as R(110)-Vo, R(110)-2Vo, respectively), as well as single Ti^3+^ with four coordination sites on anatase (101) surfaces (Fig. 2). As shown below, the number of the associated Vo’s is critical to discriminate against the active and non-active bi-Ti^3+^ sites. We utilized density functional theory (DFT) and the computational hydrogen electrode (CHE) approach^25^ that have been proven useful in understanding various electrocatalytic reactions^20,22,26–28^. More details about how to determine the active sites and the atomic configurations of different Ti^3+^ sites are further illustrated in the Methods section, and the corresponding results are presented in Fig. 3, Supplementary Fig. 1 and Supplementary Table 1.

The adsorption of N_2_ molecule is the first step to initialize the N_2_RR^22,23,29^. Both the adsorption free energy (Δ*G*) and the adsorption electronic energy (Δ*E*) are presented in Fig. 3 and Supplementary Fig. 1. As free N_2_ has a very stable triple bond, whereas the charge transfer and bond elongation are associated with high energy cost, these quantities are used as a strong evidence to show whether or not N_2_ is chemisorbed and activated. The A(101)-Vo (i.e., Vo on anatase (101) planes) presents adjacent bi-Ti^3+^ pairs, which can effectively induce the chemisorption of N_2_ in a lying-down manner and subsequent activation (Fig. 3a). This is illustrated by the fact that N_2_ molecule is 0.56 |e| charged (Bader charge^30^), which makes the N−N bond length elongated from 1.12 Å in an original N_2_ molecule to 1.18 Å in the chemisorbed state. Similar chemisorption of N_2_ was also reported on the Vo’s of anatase (010)^17^. On the contrary, the adjacent bi-Ti^3+^ pairs on R(110)-Vo (Supplementary Fig. 1a) and single Ti^3+^ sites on the A(101)-Vo (Fig. 3b) can only adsorb N_2_ in a standing-up manner, with much less charge transfer and negligible N−N bond length elongation. Furthermore, by comparing the geometry structures between the adsorbed N_2_ on R(110)-Vo and that on A(101)-Vo, it can be seen that there exists a large repulsion in the former between the adsorbed N_2_ and one of the adjacent lattice oxygens at the bridge site, owing to a close distance between negatively charged N and the lattice O^2‒^ (Fig. 2 and Supplementary Fig. 1a). Thus, it is only when such lattice O^2‒^ is removed and an adjacent bi-Ti^3+^ pair on R(110)-2Vo is formed, the efficient activation of N_2_ can occur as shown by a significant amount of negative charge on N_2_ and an elongated N−N bond length (Supplementary Fig. 1b).

Based on the aforementioned **Criterion 2**, the first hydrogenation step was further investigated. The results show that the initial N_2_ adsorption has a large impact on the subsequent hydrogenation. As illustrated in Fig. 3a and Supplementary Fig. 1b, the calculated reaction free energies (Δ*G*) for the first hydrogenation step on A(101)-Vo and R(110)-2Vo are 0.24 and 0.25 eV, respectively, much lower than those on the single Ti^3+^ sites (0.50 eV, Fig. 3b) and the adjacent bi-Ti^3+^ sites on R(110)-Vo (0.75 eV, Supplementary Fig. 1a), whereas N_2_ is also inactivated in the latter two cases. Our calculation results agree with the previous hypothesis^20^, and show that R(110)-Vo is unlikely to be the relevant surface for the N_2_RR. Here, we can now rule out the two configurations of single Ti^3+^ sites and the adjacent bi-Ti^3+^ sites on R(110)-Vo, which cannot activate N_2_ and are unfavorable for the first hydrogenation step.

By examining the **Criterion 3**, we found that the reaction free energies for the hydrogenation of NH_2_* on A(101)-Vo (Fig. 3a) and R(110)-2Vo (Supplementary Fig. 1b) are 0.18 and 0.23 eV, respectively. These numbers can be favorably compared with those for the first hydrogenation step. Hence, following the first three criteria, both the adjacent bi-Ti^3+^ pair sites on A(101)-Vo and R(110)-2Vo are active for the N_2_RR. However, as shown in Supplementary Fig. 1a and b, the formation of two adjacent Vo’s on rutile (110) surfaces is 0.36 eV higher than the formation of two separated Vo’s (see more discussion in the Methods section). This downplays the role of the adjacent bi-Ti^3+^ sites on R(110)-2Vo. Hence, the anatase (101) surface is the more suitable support for the development and enrichment of the active bi-Ti^3+^ pair sites. The feasibility of the N_2_RR on the adjacent bi-Ti^3+^ on A(101)-Vo is also confirmed by the whole free energy pathway calculated at 0 and −0.24 V as presented in Supplementary Fig. 2.

Low-valance dopants have often been utilized to facilitate the formation of Vo’s^19^. However, their incorporation into TiO_2_ does not guarantee the formation and enrichment of the active bi-Ti^3+^ sites. On the other hand, the same-valance dopants are generally useful in heterogeneous catalysis^19^, whereas their roles are less understood. Assuming that the same-valance dopants could introduce strain into the original lattice, we examined how lattice expansion and contraction would change the formation energy of Vo’s. As shown in Supplementary Fig. 3, both the tensile strain and the compressive strain can lower the formation energy of Vo’s in the anatase lattice (see the Methods for more computational details). Considering Ti^4+^ is the smallest cation for the oxidation state of M^4+^ (e.g., 72 pm of Zr^4+^, or 106 pm of Ce^4+^, as compared with 52 pm of Ti^4+^)^21^, introducing the tensile strain is practically feasible. Note that Zr^4+^ has a similar *d*-electron configuration and oxide structure, it is thus an ideal replacement of Ti^4+^. Furthermore, as the oxidation number of Zr^4+^ is fixed, the newly formed Vo’s are expected to be associated with the formation of two adjacent Ti^3+^, which are beneficial to the enrichment of the active sites. In contrast, Ce^4+^ has a much larger ion radius (106 pm), presumably lowering the formation energy of Vo’s to a larger extent. Nevertheless, it is more likely to break the original crystal structure of TiO_2_ upon Ce^4+^ doping. Furthermore, Ce^4+^ can be reduced to Ce^3+^ during the formation of Vo’s^21^, and thus does not contribute to the formation of the bi-Ti^3+^ active centers upon doping.

### Synthesis and structural characterizations

**Fig. 4:**
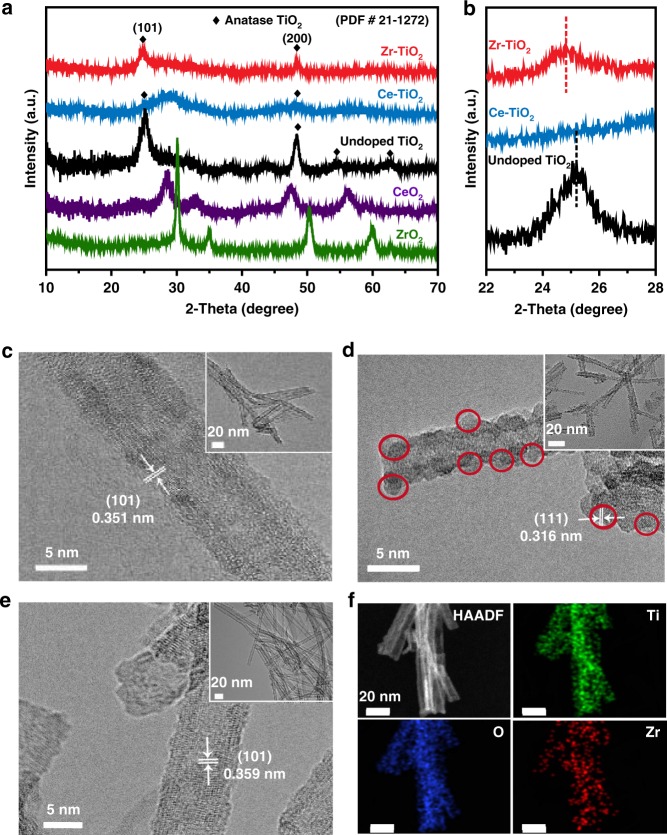
Structural and compositional characterizations. **a** X-ray diffraction (XRD) patterns of ZrO_2_, CeO_2_, undoped TiO_2_, Ce-TiO_2_, and Zr-TiO_2_. **b** The enlarged view of XRD pattern at 22^o^−28^o^ range. **c**–**e** High-resolution transmission electron microscopy (HRTEM) images of **c** undoped TiO_2_, **d** Ce-TiO_2_, and **e** Zr-TiO_2_ samples. Insets: Transmission electron microscopy (TEM) images of the corresponding nanotubes. **f** High-angle annular dark-field scanning transmission electron microscopy (HAADF-STEM) image with corresponding element mappings of Zr-TiO_2_, showing the distribution of Ti (green), O (blue), and Zr (red). (Source data are provided as a Source Data file.)

TiO_2_ anatase nanotubes were first synthesized by a hydrothermal method, followed by incubation of Zr^4+^ or Ce^4+^ dopants with subsequent annealing (see Methods section). The crystal structures of different samples were investigated by X-ray diffraction (XRD, Fig. 4a). For the undoped and Zr^4+^-doped TiO_2_ (designated as Zr-TiO_2_) nanotubes, all the diffraction peaks correspond to an anatase phase (JCPDS# 21‒1272). The TiO_2_ peak intensity decreases with the Zr^4+^ doping, suggesting the slight decrease of TiO_2_ crystallinity, but no peaks associated with ZrO_2_ are observed. Close examination of the spectra shows that with the increasing Zr^4+^ content, the XRD peaks gradually shift toward lower diffraction angles (Fig. 4b), indicating the increase of TiO_2_ lattice constants upon Zr^4+^ doping. The corresponding interplanar spacing values of the (101) planes change from 0.351 nm for undoped TiO_2_ to 0.359 nm for Zr-TiO_2_. This observation of anatase TiO_2_ lattice expansion upon the Zr^4+^ doping is supported by the DFT calculations (Supplementary Table 2 and Supplementary Table 3). In contrast, the Ce-doped TiO_2_ does not exhibit characteristic peaks of TiO_2_, but presents peaks that are associated with CeO_2_ (JCPDS# 43‒1002), suggesting the loss of TiO_2_ anatase structure and the formation of CeO_2_ crystals upon the Ce^4+^ doping. As a comparison, the Zr^4+^ doping on rutile TiO_2_ (designated as Zr-rutile-TiO_2_) was also conducted to illustrate the effect of different active sites. The XRD peaks (Supplementary Fig. 4) confirm the typical rutile phase (JCPDS# 21‒1276).

High-resolution transmission electron microscopy images show that the undoped (pristine) TiO_2_ anatase nanotubes have a tubular structure, with the outer and inner diameters of 7–9 and ~ 4 nm, respectively (Fig. 4c). The resolved lattice fringes are measured as 0.351 nm, consistent with the interplanar distance of (101) planes of anatase TiO_2_. After doping with 5% Ce^4+^ (i.e., Ce-TiO_2_, Fig. 4d), small CeO_2_ nanoparticles are observed on the exteriors of original TiO_2_ nanotubes, suggesting an inhomogeneous behavior of phase separation. In contrast, for TiO_2_ nanotubes doped with 5% Zr^4+^ (i.e., Zr-TiO_2_, Fig. 4e), no nanoparticles are observed outside the nanotubes. The resolved lattice fringes of 0.359 nm are similar but slightly larger than those of (101) planes of anatase TiO_2_, also in good accord with the XRD results. The energy-dispersive X-ray spectroscopy (EDS) and mapping confirm the existence and uniform distribution of Ti, O, and Zr elements in the nanotubes (Fig. 4f and Supplementary Fig. 5).

**Fig. 5:**
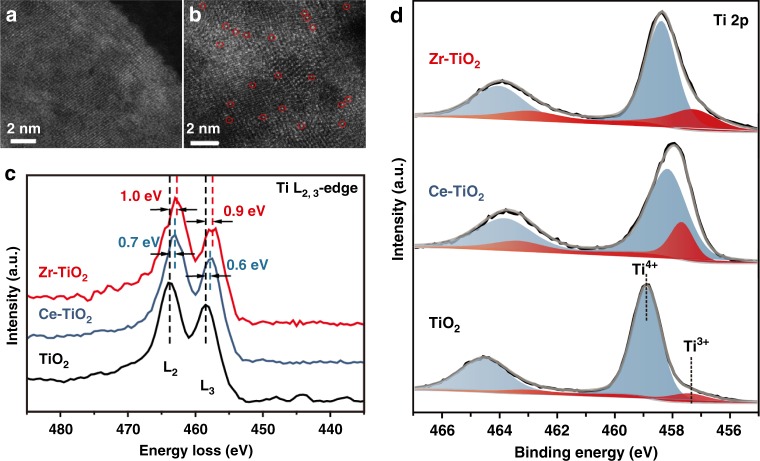
Structural and compositional characterizations. High-angle annular dark-field scanning transmission electron microscopy (HAADF-STEM) of **a** undoped TiO_2_ and **b** Zr-TiO_2_ samples. Single Zr^4+^ ions were highlighted by red circles. **c** Electron energy loss spectroscopy (EELS) profiles of the Ti-L_2, 3_ edge recorded across the undoped TiO_2_, Ce-TiO_2_, and Zr-TiO_2_ samples. **d** Ti 2p X-ray photoelectron spectroscopy (XPS) spectra of the Zr-TiO_2_, Ce-TiO_2_, and undoped TiO_2_, samples. (Source data are provided as a Source Data file.)

High-angle annular dark-field scanning transmission electron microscopy (HAADF-STEM) was then used to characterize the dopant distribution in different types of TiO_2_ nanotubes. Compared with the undoped TiO_2_ nanotubes (Fig. 5a), individual Zr^4+^ ions are observed to occupy the original positions of Ti^4+^, exhibiting a distribution of single atoms (highlighted by red circles in Fig. 5b). The electron energy loss spectroscopy (EELS) analysis of Ti-edge was conducted to probe the phase and chemical states of titanium for the undoped, Zr- and Ce-doped TiO_2_ nanotubes, respectively (Fig. 5c). The undoped TiO_2_ nanotubes present Ti^4+^ features with two peaks at 458.4 (Ti-L_3_) and 463.7 eV (Ti-L_2_), respectively. For Zr-TiO_2_, the centers of these two peaks shift to lower energy near 457.5 and 462.7 eV, respectively, indicating that the cation incorporation elevates the content of the unoccupied Ti 3*d* state as well as the distorted Ti^3+^ coordination^31^. Furthermore, a shift of 0.6 eV to lower energy is observed for the Ti-L_3_ edge in the EELS spectra of Ce-TiO_2_, associating with the existence of a distorted structure in Ce-TiO_2_ samples^31^. Based on the intensity of L_2_ and L_3_ from the Zr-TiO_2_ sample (Supplementary Fig. 6a), the ratio of Ti^3+^/∑Ti (∑Ti = Ti^3+^ + Ti^4+^) is estimated to be 29.1%^32^. Similarly, the Ti^3+^/∑Ti values of undoped TiO_2_ and Ce-TiO_2_ are calculated as 8.5% and 21.0%, respectively (Supplementary Fig. 6b, c).

X-ray photoelectron spectroscopy (XPS) was further conducted to study the oxidation states of metal ions on the catalyst surfaces. For the undoped TiO_2_ nanotubes (Fig. 5d, bottom panel), two main peaks centered at 458.9 and 464.6 eV are observed, corresponding to the Ti 2p_3/2_ and Ti 2p_1/2_ peaks, respectively. These two peaks can be further deconvoluted into four sub-peaks, assigned to Ti^3+^ 2p_3/2_ (457.4 eV), Ti^4+^ 2p_3/2_ (458.9 eV), Ti^3+^ 2p_1/2_ (463.1 eV), and Ti^4+^ 2p_1/2_ (464.6 eV), respectively, confirming the coexistence of Ti^3+^ and Ti^4+^ species^33^. The ratio between Ti^3+^ and ∑Ti for undoped TiO_2_ is calculated as 10%. The incorporation of Zr^4+^ into TiO_2_ frameworks leads to a clear shift to lower binding energy direction and widening both peaks (Fig. 5d, top panel), suggesting the increase of the Ti^3+^ content. The Ti^3+^/∑Ti ratio is calculated as 31% for Zr-TiO_2_ nanotubes, in which the increase of Ti^3+^ percentage is a clear indication of oxygen vacancy increase^34^. In comparison, the Ti^3+^/∑Ti ratio for Ce-TiO_2_ nanotubes is measured as 21% (Fig. 5d, middle panel), which can be attributed to the loss of TiO_2_ anatase structure, in good accord with XRD results. Accordingly, the analyses of both the Zr 3*d* peaks (Supplementary Fig. 7a) and Ce 3*d* peaks (Supplementary Fig. 7b) confirm the existence of Zr^4+^ and Ce^4+^/Ce^3+^ in the Zr-TiO_2_ and Ce-TiO_2_ nanotubes, respectively. The defects of unpaired electrons in materials were also probed by electron paramagnetic resonance spectra. Among the three samples (Supplementary Fig. 8), the Zr-TiO_2_ sample presents the largest signal at *g* = 2.003, further confirming its largest concentration of Ti^3+^ ions^35^.

**Fig. 6:**
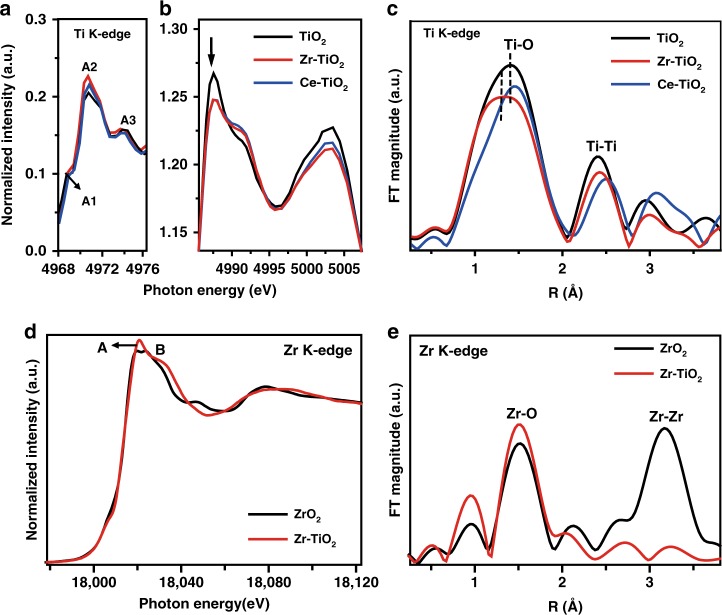
X-ray absorption spectroscopy characterizations. **a**, **b** Enlarged Ti *K*-edge X-ray absorption near-edge structure (XANES) spectra, and **c** Fourier transformed (FT) *k*^3^-weighted of Ti *K*-edge Extended X-ray Absorption Fine Structure (EXAFS) spectra of the undoped TiO_2_, Zr-TiO_2_, and Ce-TiO_2_ samples. **d** Zr *K*-edge XANES spectra and **e** Fourier-transformed *k*^3^-weighted of EXAFS spectra of pure ZrO_2_ and Zr-TiO_2_ samples. (Source data are provided as a Source Data file.)

To further verify their electronic structures, these samples were characterized by X-ray Absorption Near-edge Fine Structure (XANES) spectroscopy. The XANES spectra of Ti *K*-edge in Zr- or Ce-doped TiO_2_ samples are similar to that in undoped TiO_2_ (Supplementary Fig. 9), confirming similar local structure modification of Ti cations^36^. The main Ti pre-edge peak at 4970.9 eV (indicated by the black arrow) in the three samples (Fig. 6a) is ascribed to the weak symmetry of the surrounding Ti cations in these catalysts^37^. The slight increase of the pre-edge intensity in both Zr-TiO_2_ and Ce-TiO_2_ indicates the existence of more distorted structures and defective Ti environment^38^. The main peak of Ti *K*-edge at 4987.6 eV in Zr- or Ce-doped TiO_2_ samples (Fig. 6b, indicated by the black arrow) is lower than that in pure TiO_2_ (4987.3 eV), indicating that the Ti species are partially reduced after doping. The bond length information of different samples was further investigated by the Fourier transformed (FT) *k*^3^-weighted of Ti *K*-edge Extended X-ray Absorption Fine Structure (EXAFS) spectra (Fig. 6c). The undoped TiO_2_ nanotubes show two peaks at 1.39 and 2.41 Å, corresponding to the Ti–O and Ti–Ti bonds, respectively^39^. Interestingly, the doping of relatively larger Zr^4+^ in the TiO_2_ framework results in a contracted Ti–O bond length (1.34 Å) with a larger distribution, suggesting that Zr^4+^ cations are interstitially incorporated in TiO_2_ lattice and result in abundant surface defect sites^40^. This observation is consistent with a previous report that the contracted Ti–O bonds associated with coordinately unsaturated Ti cations acted as Lewis acid sites^41^.

The Zr *K*-edge spectra of Zr^4+^-doped TiO_2_ nanotubes were also investigated to probe the local structure surrounding Zr cations (Fig. 6d). The main Zr *K*-edge in the range of 18,020–18,040 eV is split into two peaks at 18,018.5 eV (Peak A) and 18,030.0 eV (Peak B), indicating six-coordinated Zr^4+^ cations that are consistent with the coordination of Ti^4+^ in anatase TiO_2_ structure^42^. These features are distinctively different from those of pure tetragonal ZrO_2_ with 7- or 8-coordinated Zr^4+^ cations^43^. The EXAFS spectra of Zr-TiO_2_ (Fig. 6e) only present the bond length of Zr–O (1.52 Å) but not Zr–Zr (3.20 Å), in agreement with the HAADF-STEM results that the Zr^4+^ cations in the TiO_2_ framework exhibit a single-atomic distribution.

### Electrochemical N_2_ fixation

The nitrogen temperature-programmed desorption (N_2_-TPD) was first carried out to evaluate the capability of N_2_ adsorption by these samples (Supplementary Fig. 10). The two broad peaks centered at 170 and 500 °C are attributed to physisorption and chemisorption of N_2_, respectively^44^. Both the undoped TiO_2_ and Ce-TiO_2_ show very weak chemisorption peaks. In contrast, the Zr-TiO_2_ sample presents a strong N_2_ chemisorption, suggesting that the incorporation of Zr^4+^ in the anatase TiO_2_ lattice leads to a significant increase of active sites for N_2_ adsorption.

**Fig. 7:**
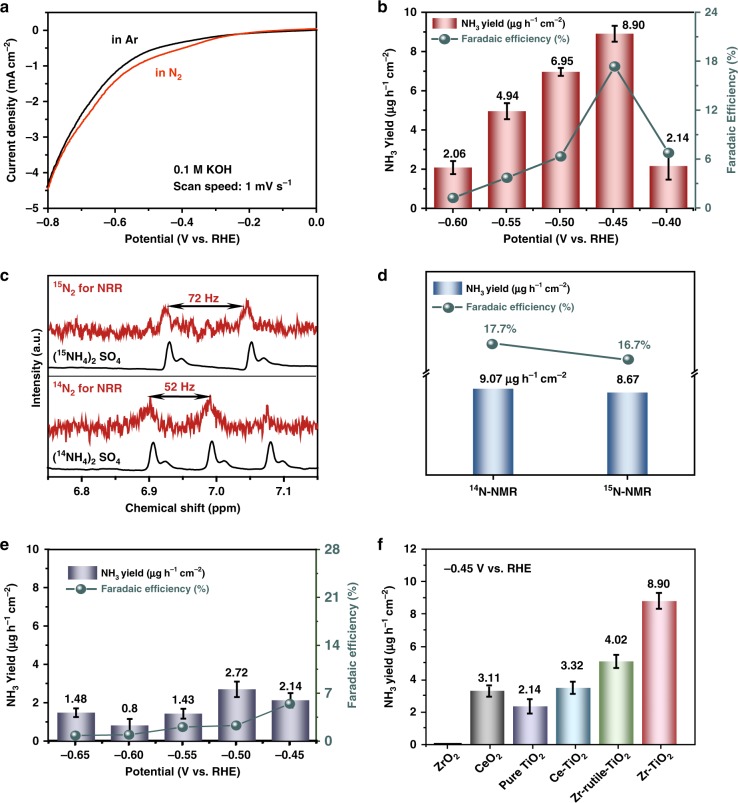
Electrochemical N_2_ fixation. **a** Linear sweep voltammetric curves in N_2_-saturated (red line) and Ar-saturated (black line) electrolytes. **b** Yield of NH_3_ production (red bars, left *y* axis) and Faradaic efficiency (green dots, right *y* axis) of Zr-TiO_2_ at each given potentials. **c** ^1^H nuclear magnetic resonance (NMR) analysis of the electrolyte fed by ^15^N_2_ (upper panel) and ^14^N_2_ (lower panel) after the electrolytic reactions. **d** Comparison of the ammonia yield rate (blue bars) and NH_3_ Faradaic efficiencies (FE_NH3_, green dots) using different feeding gases for the N_2_RR at − 0.45 V vs. RHE. **e** Yield of NH_3_ (blue bars, left *y* axis) and FE_NH3_ (green dots, right *y* axis) of undoped TiO_2_ at each given potentials. **f** Yields of NH_3_ with different catalysts at − 0.45 V vs. RHE. (Source data are provided as a Source Data file.)

The aqueous electrocatalytic N_2_ reduction was then conducted in an electrochemical cell at room temperature and pressure. N_2_ gas was supplied in a feed gas stream to the cathode, while 0.1 m KOH aqueous solution was used as the electrolyte (Methods section). All the voltages reported in this work were converted into values versus reversible hydrogen electrode (vs. RHE), as shown in Supplementary Fig. 11. The linear sweep voltammetric (LSV) curves of the Zr-TiO_2_ nanotubes were first measured in both N_2_-saturated and Ar-saturated electrolytes, respectively, in the same voltage range (Fig. 7a). A clear current density increase is observed for the N_2_-saturated electrolyte, suggesting the occurrence of the N_2_RR^11^. The thermodynamic equilibrium potential of N_2_ to NH_3_ in 0.1 m KOH is calculated as 0.056 V vs. RHE (detailed calculation shown in the Supplementary Note 1), based on the free energies tabulated in literature^45^. Here, the onset potential of the overall electrochemical reactions is defined as the total current density gets over 50 μA cm^‒2^. In order to achieve this current density, the onset potentials of the undoped TiO_2_, Ce-TiO_2_, Zr-rutile-TiO_2_, and Zr-TiO_2_ catalysts are ‒ 0.246, ‒ 0.178, ‒ 0.497, and ‒ 0.141 V vs. RHE, respectively (Supplementary Fig. 12 and Supplementary Fig. 13). For a current density of 1 mA cm^‒2^ achieved, the undoped TiO_2_, the Ce-TiO_2_, Zr-rutile-TiO_2_, and Zr-TiO_2_ require ‒ 0.643, ‒ 0.578, ‒ 0.715, and ‒ 0.538 V vs. RHE, respectively. This comparison suggests that the incorporation of Zr^4+^ should be the main contributor of active sites for catalyzing N_2_RR. The partial current densities for ammonia production were calculated by multiplying the total current density with the FE_NH3_ at selected potentials (Supplementary Fig. 14). By defining the current density level toward NH_3_ production as 25 μA cm^‒2^, the N_2_RR onset potential for the Zr-TiO_2_ is calculated as ‒ 0.4 V vs. RHE, corresponding to the overpotential of 456 mV.

The average yields of ammonia and the corresponding FE_NH3_ of those electrocatalysts were measured using the sodium salicylate-sodium hypochlorite method^13^ (Methods section). The corresponding calibration plots were displayed (Supplementary Fig. 15). All samples were measured with over three times to get the average values. The quantification of ammonia was carefully controlled to avoid possible contamination sources^46^. To rule out the possible contamination of ammonia from the air or the solution, several control experiments were carried out, as specified in Supplementary Fig. 16. Very little ammonia was detected in those controls, and the photographs of their colorimetric assays showed no color difference. In addition, as a control, the electrochemical tests were also conducted for all the electrocatalysts under Ar controls^47^. The corresponding ultraviolet-visible (UV–Vis) spectra of electrolyte after 3 h electrolysis and chromogenic reaction show the maximum values are comparable to the spectrum backgrounds (Supplementary Fig. 17), suggesting almost no ammonia was produced for all samples in an Ar-saturated electrolyte.

For the electrochemical tests of Zr-TiO_2_ conducted in N_2_-saturated electrolytes, the UV–Vis spectra show a significant enhancement of the peak centered around 660 nm, suggesting that the Zr-TiO_2_ nanotubes catalyze N_2_ reduction (Supplementary Fig. 18). No N_2_H_4_ product is detected in the electrolyte for the Zr-TiO_2_ catalysts after 3 h N_2_RR test (Supplementary Fig. 19). For the Zr-TiO_2_ nanotubes, both the ammonia production rate and the corresponding FE_NH3_ reach their peak values at − 0.45 V vs. RHE, which are calculated as 8.90 ± 0.17 µg h^−1^ cm^−2^ catalyst and 17.3% (Fig. 7b), respectively. Further increase the negative potential leads to the decrease of the ammonia production rate and FE_NH3_, which can be attributed to the increase of the competitive HER on the electrode surfaces. The quantitative measurement of the ammonia production was further verified by two other methods, including the ion chromatography (IC) and the nuclear magnetic resonance (NMR)^48^. The ^1^H NMR spectra show a triplet coupling (~ 52 Hz) for ^14^NH_4_^+^ and a doublet coupling (~ 72 Hz) for ^15^NH_4_^+^ (Fig. 7c). The obtained ammonia production rate and the corresponding FE_NH3_ for ^15^N_2_ as the feeding gas are comparable to those used ^14^N_2_ (Fig. 7d and Supplementary Fig. 20), confirming that the ammonia detected is attributed to the electroreduction of N_2_. The total ammonia produced during the 3 h electrochemical reaction time was calculated as 0.538 μmol (detailed calculation shown in the Supplementary Note 2).

In contrast, all the undoped TiO_2_ (Fig. 7e), Zr-rutile-TiO_2_ (Supplementary Fig. 21), and Ce-TiO_2_ (Supplementary Fig. 22) electrocatalysts exhibit much lower ammonia production rates, with the peak values of 1.48, 3.22, and 5.79 µg h^−1^ cm^−2^ catalyst at a higher negative potential of − 0.65 V, respectively. Furthermore, the N_2_RR tests were also conducted on ZrO_2_ and CeO_2_ nanoparticles under the same catalytic potential of − 0.45 V vs. RHE to probe the effect of Zr-incorporation (Fig. 7f). Both the pure CeO_2_ and ZrO_2_ nanoparticles exhibit lower or even negligible NH_3_ production rates. This comparison suggests that the bi-Ti^3+^ pairs induced by Zr^4+^-doped TiO_2_ function as excellent electrocatalytic centers for the N_2_ fixation at ambient conditions.

Finally, the chronoamperometry tests at selected applied potentials of − 0.5 and − 0.45 V (where the ammonia yields were the highest) show that the N_2_RR performance of the Zr-TiO_2_ nanotubes catalyst was stable after several hours of continuous electrolysis (Supplementary Fig. 23). This cycling stability test was further repeated for a total of six runs (Supplementary Fig. 24), showing no obvious change in the NH_3_ yield rate and current efficiency. After the electrochemical test, the Zr-TiO_2_ catalyst was re-measured with XPS (Supplementary Fig. 25). No obvious difference is observed compared with that before the electrochemical test, further confirming the good stability of the Zr-TiO_2_ nanotubes in N_2_RR electrocatalysis.

In this work, we have first screened several types of Ti^3+^ sites by means of DFT calculations. The adjacent bi-Ti^3+^ pairs formed on the most-stable surface of anatase TiO_2_ (i.e., A(101)-Vo) are identified as the active electrocatalytic centers, which can lead to a lying-down manner as efficient N_2_ chemisorption and subsequent activation. However, similar bi-Ti^3+^ pair sites formed on the most stable surface of rutile TiO_2_ (i.e., R(110)-Vo), as well as the single Ti^3+^ site, are concluded as the inactive sites.

By further removing a second O^2‒^ to form R(110)-2Vo, the adjacent bi-Ti^3+^ pairs on rutile TiO_2_ can now induce a lying-down chemisorption manner for N_2_ (Supplementary Fig. 1b), exhibiting a significant amount of negative charge on N_2_ and an elongated N−N bond length. Nevertheless, as shown in Supplementary Fig. 1a and b, the formation of two adjacent Vo’s on rutile (110) surfaces is 0.36 eV higher than the formation of two separated Vo’s, downplaying the role of the adjacent bi-Ti^3+^ sites on R(110)-2Vo. Hence the anatase (101) surface is the more suitable host for the development and enrichment of the active bi-Ti^3+^ pair sites.

The competitive adsorption of H_2_O and activation of HER on these bi-Ti^3+^ catalytic sites are also estimated (Supplementary Table 4). For convenience, here we assume the chemical potential of the water in solution is equal to 3.169 kPa as pure liquid water at room temperature. Then, the calculated adsorption free energy changes is only 0.04 eV, when the adsorbed H_2_O on the bi-Ti^3+^ sites is replaced by the N_2_ under working condition (the corresponding structures are presented in Supplementary Fig. 26). For HER, the adsorption free energy of the first hydrogen is calculated to be 0.19 eV on the oxygen vacancy, which is only slightly more advantageous than that of N_2_RR of 0.24 eV. Meanwhile, one has to take into account the fact that the high pH of the electrolyte (pH 13) has an inhibiting effect on the HER^46^. Thus, N_2_ in our situation is able to compete with H_2_O and H for adsorption and activation as shown in our experiments.

In addition, previous literatures show that if the anatase nanoparticles are exposed under reduction situation with hydrogen, it results in reduced nanoparticles comprising a crystalline TiO_2_ core and a disordered shell with abundant oxygen vacancies^49^. With respect to the corresponding pristine surface O*, the calculated free energy diagrams for the further reduced surface OH* and H_2_O* of pristine A(101) surface, with and without Zr-doping under different potentials, are shown in Supplementary Fig. 27. The result suggests that the surface with O* totally hydrogenated is the most stable under working conditions for *U*_RHE_ < −0.40 eV, such that the oxygen vacancies should remain thermodynamically stable, which should not be annihilated by the O** or OH* species.

As Zr^4+^ is not a reducible cation, it is not expected to directly form Vo’s next to Zr^4+^, but our work shows that the doping of Zr^4+^ into the anatase TiO_2_ framework is a useful strategy to induce and enrich the specific adjacent bi-Ti^3+^ pairs on the anatase surfaces. Owing to its similar *d*-electron configuration and oxide structure but relatively larger ionic size as compared with Ti^4+^, the doped single Zr^4+^ ion induces a strained effect without breaking the original TiO_2_ structure, which, in turn, enhances the formation of oxygen vacancy and subsequently bi-Ti^3+^ sites on the anatase surfaces. Control experiments reveal that Ce^4+^ doping does not have the same role as Zr^4+^ doping, for Ce^4+^ has a too larger size and a variable oxidation state.

Based on an expanded anatase lattice with lattice constants that are 1.023 times larger than the optimized one by DFT, various anatase A(101) surfaces were built without or with different Zr^4+^ concentrations in surfaces or subsurfaces. The calculated results for some representatives of the strained A(101) surfaces are shown in Supplementary Table 3. The Vo formation energy without Zr^4+^ doping is calculated to be 0.27 eV lower than the A(101) surface based on the optimized lattice. With various contents of Zr^4+^ dopants in surfaces or subsurfaces, the calculated Vo formation energies are within 0.22 and 0.33 eV smaller than that of the optimized lattice. The DFT results support the experiment observation that Zr^4+^ enhances the Vo formation on A(101) surface, which is owing to the tensile strain induced by the Zr^4+^ doping.

Owing to the efficient chemisorption and activation of the N_2_ molecules by the Zr^4+^ doping-induced bi-Ti^3+^ pairs on anatase TiO_2_, the Zr-TiO_2_ exhibits an outstanding ammonia production rate, a high FE_NH3_, and excellent electrochemical stability, significantly exceeding those of the undoped TiO_2_ or Ce-TiO_2_ samples under similar testing conditions. Compared with other N_2_RR electrocatalysts in aqueous solutions at ambient conditions reported to date (Supplementary Table 5), our Zr-TiO_2_ sample demonstrated one of the highest NH_3_ production rates and FE_NH3_ values.

In conclusion, our study demonstrates the bonding nature of the active centers and a unique approach of optimizing electrocatalytically active sites by rational design of dopant size and charge, enabling new opportunities for efficient electrochemical N_2_ reduction. Our work not only reveals bi-Ti^3+^ pairs on anatase TiO_2_ as effective N_2_RR active centers, but also suggests an attractive viewpoint to understand and apply the same-valance dopants in heterogeneous catalysis, which is generally useful but still poorly understood. Further development of similar multiple active sites with cooperative binding and activation effects on N_2_ may lead to a vast variety of opportunities of enhancing the N_2_RR capabilities and potential large-scale utilization toward direct atmospheric N_2_ fixation.

## Methods

### Chemicals and materials

Titanium (IV) oxide (P25, Sinopharm Chemical Reagent Co., Ltd, analytically pure), zirconium nitrate pentahydrate (Aladdin, Z190748, 99.5%), cerium nitrate hexahydrate (Aladdin, C105378, 99.5%), sodium hydroxide (Macklin, S817977, ≥ 98%), salicylic acid (Macklin, S817529, 99.5%), potassium sodium tartrate tetrahydrate (Macklin, P816438, 99.5%), sodium nitroferricyanide(III) dehydrate (Macklin, S818341, 99.98% metals basis), sodium hypochlorite solution (Macklin, S828471, available chlorine 4.0%), ammonium chloride (Maclin, A801305, 99.8%), hydrazine monohydrate (Alfa Aesar, A14005, 98%), hydrochloric acid (Sinopharm Chemical Reagent Co., Ltd, 10011018, 36.0−38.0%), potassium hydroxid (Aladdin, P112287, 99.99% metals basis), sulfuric acid (Sinopharm Chemical Reagent Co., Ltd, 10021618, 95.0−98.0%), ethyl alcohol (Sinopharm Chemical Reagent Co., Ltd, 10009218, ≥ 99.7%), 4-(dimethylamino) benzaldehyde (Sigma-Aldrich, 156477, 99%), Nafion solution (Dupont), 211 Nafion membrane (Dupont), deionized (DI) water (Millipore, 18.2 MΩ cm), N_2_ gas (99.99%), Ar gas (99.99%). Ammonium sulfate ((NH_4_)_2_SO_4_, 99%), (^15^NH_4_)_2_SO_4_, 98 atom% ^15^N), and nitrogen-^15^N_2_ (98 atom% ^15^N) were purchased from Sigma-Aldrich. All chemical reagents were used as received without further purification.

### Characterization

The XRD data were characterized by Bruker SMART APEX (II)-CCD (Germany). X-ray photoelectron spectroscopy was recorded on a Perkin Elmer PHI 5000 C ESCA system (Perkin Elmer, USA). The high-resolution transmission electron microscopy images and the EDX spectroscopy spectra were recorded by a JEM 2100 F (JEOL, Japan) and a Tecnai T20 (FEI, USA) transmission electron microscope. The HAADF-STEM, EELS, and the EDX mapping experiments were performed using Titan Cubed Themis G2 300 (FEI) microscope equipped with Super-X detectors at 200 kV. N_2_-TPD measurements were performed on a Micrometrics Autochem II 2920 system. Electron-spin resonance signals were recorded on a Bruker ESR A300 spectrometer at room temperature. XANES and EXAFS data were collected on beamline 14 W at the Shanghai Synchrotron Radiation Facility (SSRF). The UV–Vis absorption spectrum was recorded by an ultraviolet-visible spectrometer (U-3900H, Hitachi, Japan). ^1^H-NMR (nuclear magnetic resonance) measurements were performed on a Bruker NMR600. IC analysis was performed on an ICS-2000 (Thermo Fisher Scientific) equipped with an isocratic pump.

### Synthesis of Zr-TiO_2_

In a typical synthesis, 2.0 g of TiO_2_ (P25) was mixed with 60 mL of (10 m) NaOH solution in a Teflon-lined stainless autoclave at 150 °C for 20 h. The slurry was washed with 0.1 m HCl solution for several times until the pH value reached 1.6, and then with DI water until pH was close to 7, before being filtrated to obtain TiO_2_ nanotubes. The introduction of Zr^4+^ to TiO_2_ was conducted by wet impregnating the TiO_2_ nanotubes with 20 mL of 0.35 m zirconium nitrate solution. The mixture was stirred at room temperature for 4 h. After the reaction, the substrate was washed with DI water and ethanol for several times, followed by drying at 60 °C and then annealed in Ar at 400 °C, with a ramping rate of 2 °C min^‒1^ for 2.5 h.

### Electrochemical measurements

A total of 5 mg of catalyst was dispersed in 0.5 mL of ethanol followed by the addition of 50 µL of Nafion solution. The mixture was sonicated thoroughly to form a homogeneous ink. The working electrodes were then prepared by drop-casting the catalyst inks onto carbon paper to achieve a loading of 1.0 mg cm^‒2^. All the electrochemical performance measurements were performed with an Autolab electrochemical workstation (Autolab PGSTAT204) at room temperature (25 ± 2 °C) using 0.1 m KOH as the electrolyte. For the electrocatalytic N_2_RR, a saturated calomel electrode (SCE) and a Pt wire were used as reference and counter electrodes, respectively. The potentials were all converted to the RHE scale according to Nernst equation (1):

1 $$E_{{\mathrm{RHE}}}\left( {\mathrm{V}} \right) = E_{{\mathrm{SCE}}}\left( {\mathrm{V}} \right) + E^o_{{\mathrm{SCE}}}\left( {\mathrm{V}} \right) + 0.0591 \times {\mathrm{pH}} = E_{{\mathrm{SCE}}}\left( {\mathrm{V}} \right) + {\mathrm{1}}{\mathrm{.008}}\;{\mathrm{V}}$$

where *E*_RHE_ is the converted potential vs. RHE, *E*_SCE_ is the experimental potential measured vs. SCE, *E*^*0*^_SCE_ is the standard potential of SCE at 25 °C. The potential of SCE was calibrated to the RHE in 0.1 m KOH electrolyte saturated with high-purity H_2_ (Supplementary Fig. 11), consistent with the Equation (1). The scan rate for LSV was kept at 1.0 mV s^‒1^.

For the potentiostatic measurement, the KOH electrolyte (pH 13, 30 mL) was purged with pure N_2_ for 30 min before the measurement. For comparison, the same electrochemical test was also conducted in an Ar-saturated KOH solution. Constant potential electrolysis was conducted at various potentials for 3 h. The electrochemical experiments were repeated for three times to obtain the averaged measured values.

### Determination of ammonia

The concentration of produced ammonia was spectroscopically determined by the indophenol blue method^50^ with some modification. Sodium salicylate (5 g), sodium hydroxide (1.47 g), and potassium sodium tartrate tetrahydrate (5 g) were dissolved in DI water and diluted to 100 mL was used as the color reagent A. In brief, 8 mL of the testing electrolyte was taken from the electrochemical cell, followed by adding 1 mL of color reagent A, 100 μL of (10 mg/mL) sodium nitroferricyanide (C_5_FeN_6_Na_2_O), and 100 μL of 0.05 m NaClO. The UV-Vis absorption spectra were then measured. The concentration of indophenol blue was determined using the absorbance at 660 nm. The concentration-dependence absorption curves were calibrated using standard ammonia chloride solutions with different concentrations. All the spectroscopic measurements were repeated for three times to obtain the averaged measured values. The fitting curve (*y* = 0.1066*x* + 0.0109, *R*^2^ = 0.9991) showed good linear relation of absorbance value with NH_3_ concentrations.

### Determination of NH_3_ yield rate and *FE*_NH3_

The electrochemical ammonia yield rate (*r*_NH3 (electrochemical)_) was calculated by subtracting the background signal of non-electrochemical ammonia, using Equation (2):

2 $$r_{{\mathrm{NH3}}\;\left( {{\mathrm{electrochemical}}} \right)} = \frac{{\Delta c_{{\mathrm{NH}}_{{\mathrm{3}}\;{\mathrm{(yield)}}}} \times V_{{\mathrm{aq}}}}}{{t \times A}}$$

where Δ*c*_NH3 (yield)_ = *c*_NH3 (yield, N2)_−*c*_NH3 (yield, Ar)_.*c*_NH3 (yield, N2)_ is the measured NH_4_^+^ mass concentration in an N_2_-saturated electrolyte, *c*_NH3 (yield, Ar)_ is the measured NH_4_^+^ mass concentration in an Ar-saturated electrolyte as a control. *V*_aq_ is the volume of electrolyte, *t* is the electrochemical reaction time, and *A* is the geometric area of the cathode.

The electrochemical ammonia faradaic efficiency (*FE*_NH3 (electrochemical)_) was calculated by subtracting the background signal of non-electrochemical ammonia^47^, using Equation (3):

3 $${\mathrm{FE}}_{{\mathrm{NH3}}\;\left( {{\mathrm{electrochemical}}} \right)} = \frac{{n \times {\mathrm{\Delta c}}_{{\mathrm{NH}}_{_{{\mathrm{3}}\;{\mathrm{(yield)}}}}} \times V_{{\mathrm{aq}}} \times F}}{{{\mathrm{17}} \times Q}} \times {\mathrm{100}}\;{\mathrm{\% }}$$

where *n* is the number of transferred electrons (3), *F* is the Faraday constant, and *Q* is the quantity of applied electric charges.

In order to identify the source of ammonia, ^15^N_2_ enriched gas was employed to serve as the feeding gas. After N_2_ reduction reaction, the NH_4_^+^-containing electrolyte was characterized by ^1^H NMR measurement, using 1 mm maleic acid as an internal standard. 10% dimethyl sulphoxide (DMSO)-*d*_*6*_ was used as the solvent. A doublet coupling (~ 72 Hz) for ^15^NH_4_^+^ and a triplet coupling (~ 52 Hz) for ^14^NH_4_^+^ were observed for different testing solutions.

For the IC method, 3 mL of electrolyte was transferred into the chromatograph. The sample loop was 20 μL, with methanesulfonic acid as the eluent. The concentration-dependence plot by the IC method was characterized with different standard NH_4_^+^ concentrations.

Note that specific precautions were necessary with rigorous control experiments to minimize possible ammonia contamination during the N_2_RR test^46^. (1) The chemicals including water were purchased or prepared with high purity. (2) All the electrochemical measurements including control experiments were conducted at fume hoods to provide a clean atmosphere. (3) Considering the effect of human respiration and skin contaminates, masks and latex gloves (pre-soaked in 100 mL of 0.01 m HCl for 3 h) were used during the N_2_RR tests and ammonia quantification measurement.

### Determination of hydrazine

The hydrazine presented in the electrolyte was estimated by the method of Watt and Chrisp^51^. A mixture of para-(dimethylamino) benzaldehyde (5.99 g), HCl (concentrated, 30 mL) and ethanol (300 mL) was used as a color reagent B. In brief, 5 mL of testing electrolyte after the electrocatalytic reaction was taken out from the electrochemical cell, followed by adding 5 mL of color reagent B. After 20 min for color development, the UV-Vis absorption spectra were measured at 455 nm. The concentration-dependence plot was obtained by using standard hydrazine monohydrate solutions with different concentrations. The fitting curve showed good linear relation of absorbance with N_2_H_4_·H_2_O concentration (*y* = 0.7624*x* + 0.0161, *R*^2^ = 0.9992) by three times independent calibrations.

### Computational details

The Vienna ab initio simulation package was utilized to perform all DFT calculations^52–54^. The 2 *s*, 2*p* electrons in oxygen and nitrogen, the 3*d*, 4 *s* electrons in titanium and the 5 *s*, 4*d*, 5*p* electrons in zirconium were treated as valence electrons, whereas the kinetic energy cutoff for the plane wave basis sets was set to be 400 eV. The remaining core electrons were described by the projector augmented-wave method^55^. The surface Monkhorst–Pack meshes^56^ of 2 × 2 × 1 and 5 × 5 × 5 *k*-point sampling in the surface Brillouin zone were employed for slab model and bulk, respectively. For bulk optimization, all atoms as well as lattice constants were allowed to fully relax. For systems involving anatase (101), a 1 × 3 supercell of 12 atomic layers was used, where the bottom five layers of atoms were fixed in their optimized bulk positions, whereas the top seven layers, as well as the adsorbate, were allowed to fully relax. For systems involving rutile (110), a 3 × 2 supercell of 12 atomic layers was used, where the bottom five layers of atoms were fixed in their optimized bulk positions, whereas the top seven layers, as well as the adsorbate, were allowed to fully relax. After the convergence criteria for optimizations were met, the largest remaining force on each atom was less than 0.02 eV Å^‒1^. For all calculations, the spin polarized generalized gradient approximation of the Perdew–Burke–Ernzerhof functional^57^ was used. As the standard DFT functions tended to over-delocalize electrons, DFT + *U* was employed^58^ with an effective *U* value of 3.3 eV for Ti 3*d*-orbitals, as obtained from linear response^59^. For the dopants, we used standard DFT on Zr, as there were no issues with describing the electronic structure^21^. For surface reactions, the contributions of dispersive interactions were accounted for by using the DFT + D3 method with Becke-Jonson damping^60,61^. Bader’s theory of atoms in molecules was used for charge analysis^30,62,63^.

Oxygen vacancy formation energies were calculated as:

4 $$\Delta E\left( {{\mathrm{Vo}}} \right) = E_{\mathrm{t}}\left( {{\mathrm{Ti}}_{\mathrm{x}}{\mathrm{O}}_{{\mathrm{2x-1}}}} \right) + 1/2\,E_{\mathrm{t}}\left( {{\mathrm{O}}_2} \right)-E_{\mathrm{t}}\left( {{\mathrm{Ti}}_{\mathrm{x}}{\mathrm{O}}_{{\mathrm{2x}}}} \right)$$

where *E*_t_(Ti_x_O_2x-1_) and *E*_t_(Ti_x_O_2x_) are the total energies of the optimized supercell with and without V_O_ vacancy, respectively, and *E*_t_(O_2_) is the total energy of a gas phase O_2_. The second Vo formation energies, from Ti_x_O_2x-1_ were calculated as:

5 $$\Delta E\left( {{\mathrm{Vo}}} \right) = E_{\mathrm{t}}\left( {{\mathrm{Ti}}_{\mathrm{x}}{\mathrm{O}}_{{\mathrm{2x-2}}}} \right) + 1/2\,E_{\mathrm{t}}\left( {{\mathrm{O}}_{\mathrm{2}}} \right)-E_{\mathrm{t}}\left( {{\mathrm{Ti}}_{\mathrm{x}}{\mathrm{O}}_{{\mathrm{2x-1}}}} \right)$$

where *E*_t_(Ti_x_O_2x-2_) is the total energies of the optimized supercell with the first and second V_O_ vacancy, respectively. This type of oxygen vacancy formation energy is commonly used in theoretical heterogeneous catalysis, as it is convenient to compare different oxides (doped or undoped) under different reaction conditions^18^. Indeed, the formation of oxygen vacancies in reducible oxides like TiO_2_ and CeO_2_ is relatively easy^18^, in particular, under the reduction conditions such as H_2_ or CO atmosphere, or on the anode surfaces as in the present work. The formation energy of oxygen vacancies should be related with H_2_O or CO_2_ formation, thus providing the thermodynamic driving force.

The adsorption free energies of gases on the surfaces were calculated as:

6 $$\Delta G\left( {{\mathrm{gas}}} \right) = G_{\mathrm{t}}\left( {{\mathrm{gas/surface}}} \right)-G_{\mathrm{t}}\left( {{\mathrm{gas}}} \right)-G_{\mathrm{t}}\left( {{\mathrm{surface}}} \right){\mathrm{,}}$$

where *G*_t_(gas/surface), *G*_t_(surface), and *G*_t_(gas) are the total free energies of the adsorption systems of surfaces with or without vacancies, and adsorbate species in the gas phase, respectively. Here, we assumed that in addition to the total electronic energies, only the translation and rotation contributions of the gas phase species are significant and the other parts can be ignored. Assuming the gas phase species as ideal gases, the partition functions of translation *Q*^trans^ and rotation *Q*^rot^ were calculated as:^64^

7 $$Q_{\mathrm{A}}^{{\mathrm{trans}}} = \left( {\frac{{{\mathrm{2\pi }}m_{\mathrm{A}}k_{\mathrm{B}}T}}{{h^2}}} \right)^{\frac{3}{2}}{\mathrm{V}}$$

8 $$Q_{\mathrm{A}}^{{\mathrm{rot}}} = \frac{1}{\sigma }\left( {\frac{{k_{\mathrm{B}}{\mathrm{T}}}}{h}} \right)^{{\mathrm{3/2}}}\sqrt {\frac{{\mathrm{\pi }}}{{A^{{\mathrm{rot}}}B^{{\mathrm{rot}}}C^{{\mathrm{rot}}}}}}$$

where *P* (1 atm) and *m* are the partial pressure and molecular mass, respectively, *k*_*B*_ is the Boltzmann constant, *T* (298.15 K) is the absolute temperature, $V = \frac{{k_{\mathrm{B}}T}}{{P_{\mathrm{A}}}}$ is the volume of the system, *σ* is the symmetry factor, *A*^rot^, *B*^rot^, *C*^rot^ are rotational constants, and *h* is the Plank’s constant.

In our work, the DFT calculations were carried out to rationally screen four types of Ti^3+^ for a better understanding of bonding structures of the active centers (Fig. 2): (a) the adjacent bi-Ti^3+^ on anatase (101) surfaces with one oxygen vacancy (i.e., A(101)-Vo); (b) the single Ti^3+^ with four coordination on anatase (101) surfaces with one oxygen vacancy; (c) the adjacent bi-Ti^3+^ on rutile (110) surfaces with one oxygen vacancy, (i.e, R(110)-Vo); (d) the adjacent bi-Ti^3+^ on rutile (110) surfaces with a pair of oxygen vacancies together, (i.e., R(110)-2Vo’s). Cases (a) and (c) were chosen because anatase and rutile are the most common crystal phases for TiO_2_, and anatase (101) and rutile (110) surfaces are, respectively, the most exposed surfaces. Case (b) was chosen for its low coordination. Case (d) was formed after the removal of one nearby lattice O^2‒^ in Case (c). This nearby lattice O^2‒^ presents a large repulsion with the chemisorbed N_2_ in a lying-down manner, which precludes the bi-Ti^3+^ site of R(110)-Vo from effective N_2_ activation.

The overall N_2_RR process (N_2_ + 6 H^+^ + 6e^‒^ → 2NH_3_) involves several proton-coupled electron transfer steps. The Gibbs free energy change (Δ*G*) of each elementary step was calculated by using the standard hydrogen electrode model^27,28,65^, which uses one-half of the chemical potential of hydrogen as the chemical potential of the proton-electron pair. According to this method, the Δ*G* value can be determined as:

9 $$\Delta G = \Delta H-T\Delta S + \Delta G_{\mathrm{U}} + \Delta G_{{\mathrm{pH}}}$$

where Δ*H* and Δ*S* are the enthalpy change and entropy change, respectively. Δ*G*_U_ is the free energy contribution related to electrode potential U. Δ*G*_pH_ is the correction of the H^+^ free energy by the concentration, which can be calculated as

10 $$\Delta G_{{\mathrm{pH}}} = 2.303 \times k_B{\mathrm{T}} \times {\mathrm{pH}}$$

## Supplementary information

Supplementary Information

Source Data

## Data Availability

All data relevant to this study are available from the corresponding author upon reasonable request. The source data underlying Fig. 3–7 and Supplementary Figs. 4–25 are provided as a Source Data file.
