# Supplementary material for: Doping strain induced bi-Ti3+ pairs for efficient N2 activation and electrocatalytic fixation
Source: Nat Commun. 2019 Jun 28;10:2877. doi: 10.1038/s41467-019-10888-5 (PMC6599206; doi:10.1038/s41467-019-10888-5)
Supplement: Supplementary file 1 — Supplementary Information [file 41467_2019_10888_MOESM1_ESM.pdf]

Cao et al.

Doping Strain Induced bi-Ti^{3+} Pairs for Efficient N_2 Activation and Electrocatalytic Fixation

Supporting Information

Supplementary Figures

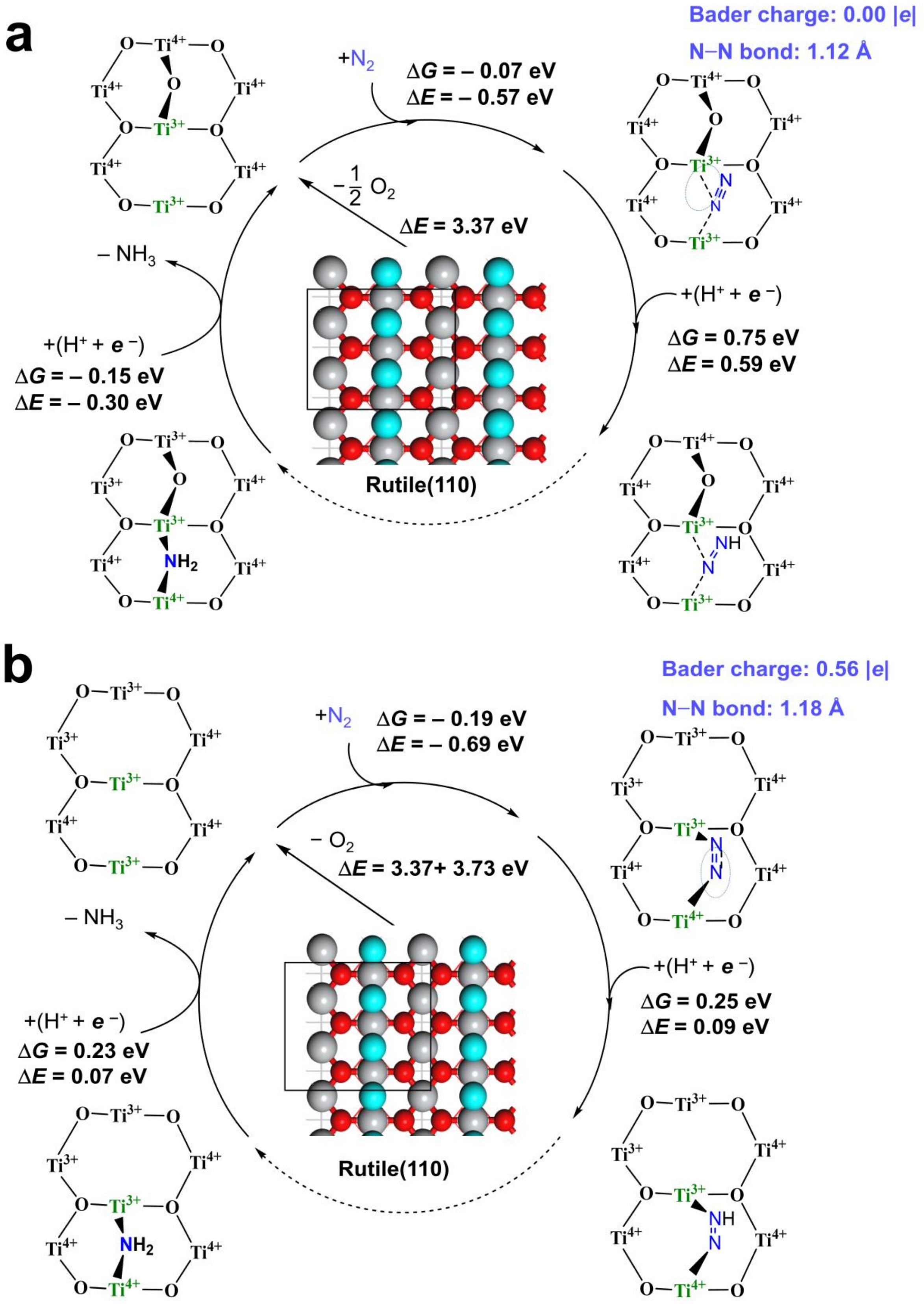

Supplementary Figure 1. DFT calculations following the four criteria for different types of Ti^{3+} site. a adjacent bi-Ti^{3+} on rutile (110) surface with one oxygen vacancy (i.e., R(110)-Vo), **b** adjacent bi-Ti^{3+} on rutile (110) surface with two adjacent oxygen vacancies (i.e., R(110)-2Vo). The light blue spheres stand for the lattice oxygens at the bridge sites where the surface oxygen vacancies are formed most easily. The red spheres stand for the other lattice oxygens on the surfaces and the grey spheres stand for the titanium cations. As compared (**a**) and (**b**), the bridge lattice oxygen, which would exert a large repulsion with the chemisorbed N_2 if it adopted a lying-down mode, is circled in blue. Here ΔG refers to the free energy, and ΔE refers to the electronic energy. The Vo formation energies, ΔE(Vo) and ΔE(2Vo), are calculated in related to the 1/2 O_2 formation as **Equation 4** and **5** in the Methods section. The formation of two adjacent Vo's on rutile (110) surfaces is (3.73 − 3.37) = 0.36 eV higher than the formation of two separated Vo's.

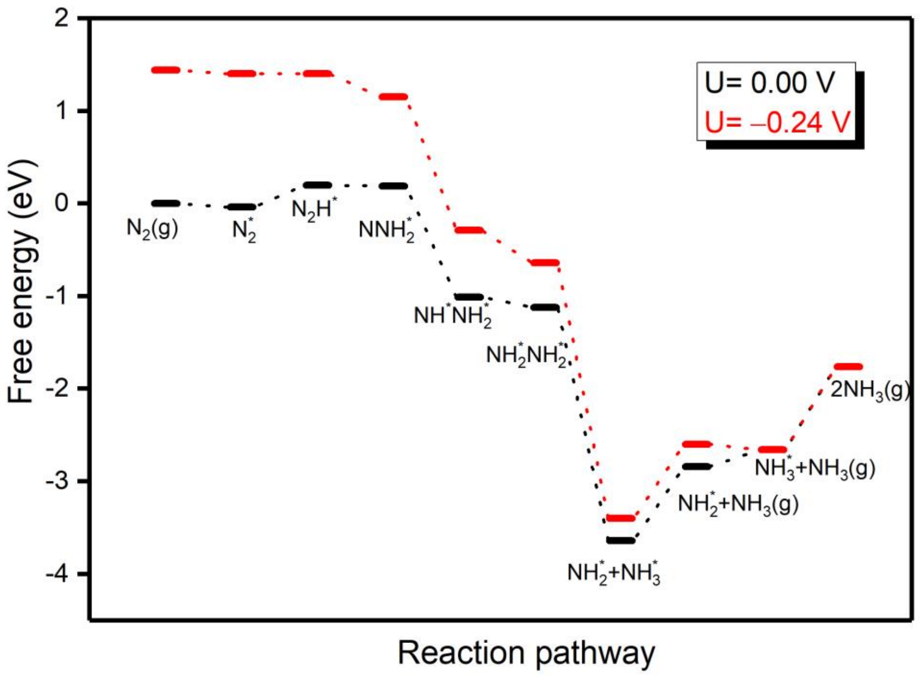

Supplementary Figure 2. Free energy diagrams of the N_2RR on the adjacent bi-Ti^{3+} on A(101)-Vo. The results were obtained at zero and limiting potentials, and the (NH_3(g) pressure was set as 1 atm).

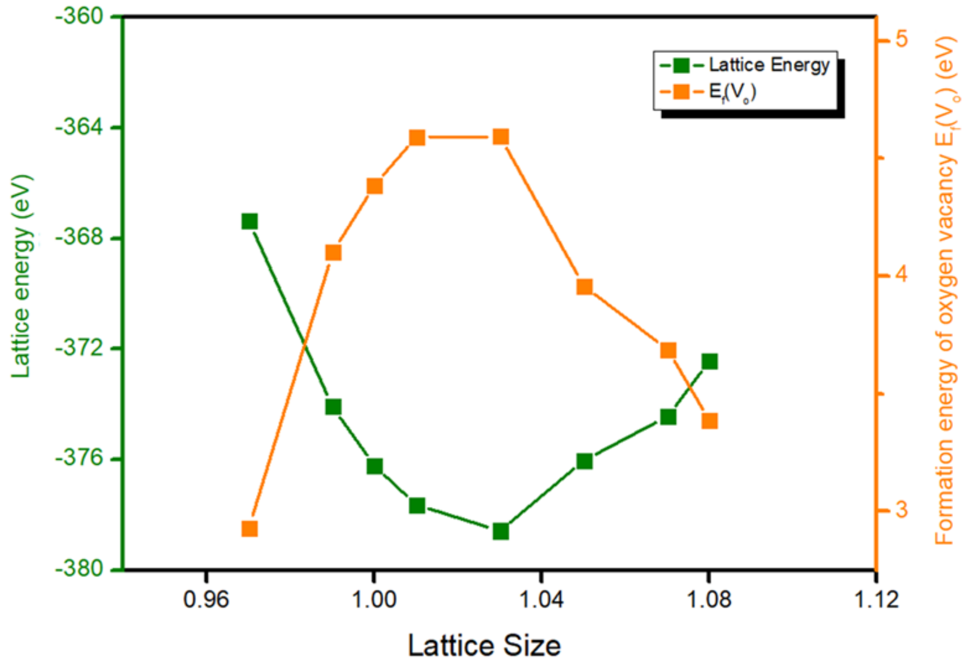

Supplementary Figure 3. The influence of anatase lattice size on the formation energy of oxygen vacancy. Total energies of the lattices (left) and the corresponding oxygen vacancy formation energies (right) for anatase TiO_2 (2 x 2 x 1 supercell) plotted against the lattice sizes relative to the lattice constants of experiment (a = b = 3.776 Å, c = 9.486 Å). The lattice constants a, b and c are strained at the same ratio. The present DFT calculations yield lattice constants which are around 1.03 times larger than the experimental ones. From the computational results, there is a tensile strain when the lattice constants are larger than the optimized ones; while there is compressive strain when the lattice constants are smaller than the optimized ones. Here, the lattice constants are fixed and only the atoms are fully relaxed during each optimization.

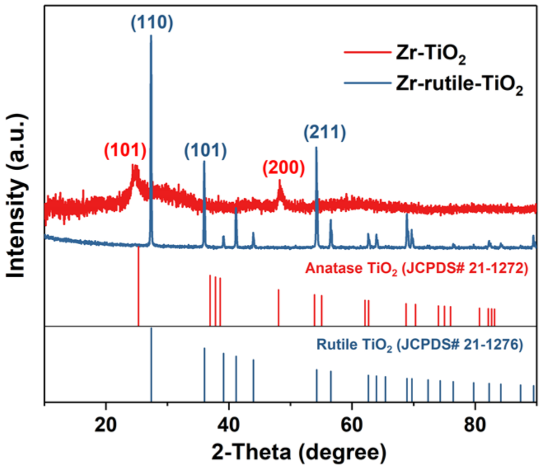

Supplementary Figure 4. XRD characterizations of the catalysts. XRD patterns of Zr-TiO_2, Zr-rutile-TiO_2 samples. (Source data are provided as a Source Data file.)

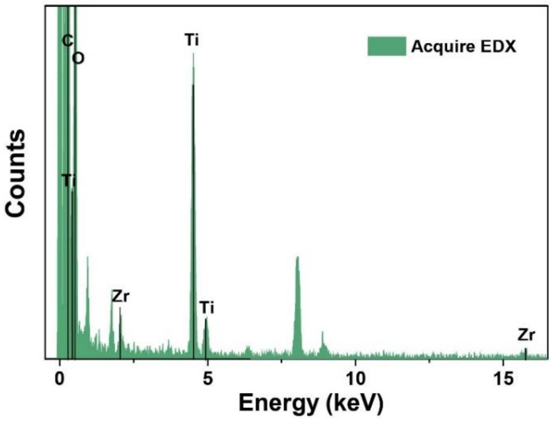

Supplementary Figure 5. The EDX spectrum of the Zr-TiO_2 sample. (Source data are provided as a Source Data file.)

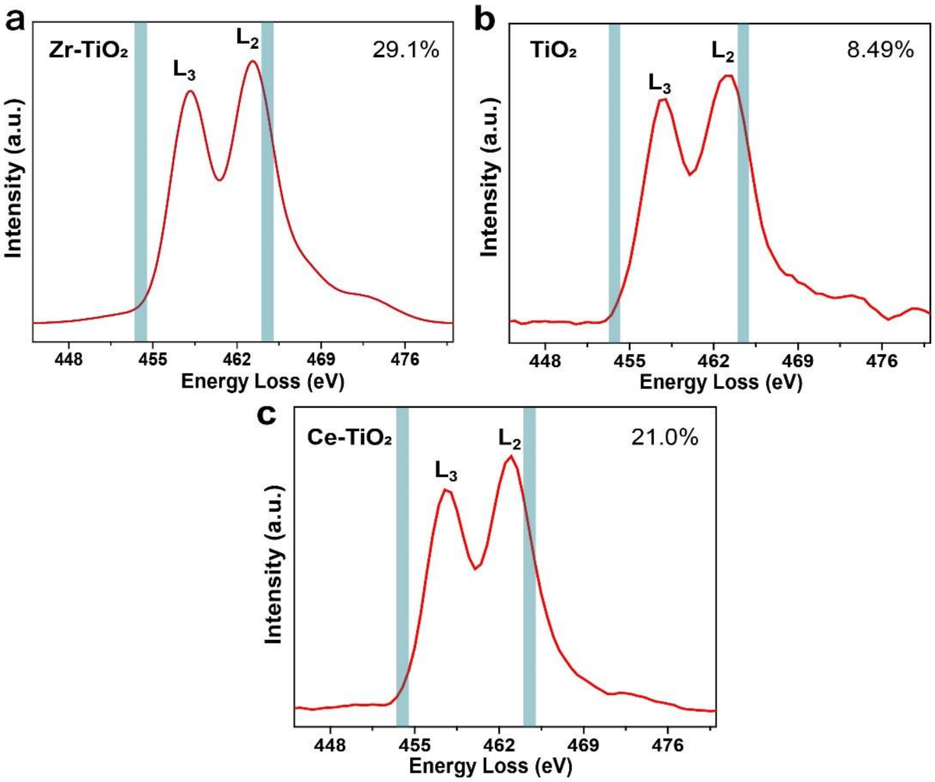

Supplementary Figure 6. EELS-STEM spectra of the catalysts. EELS-STEM spectra of **a** Zr-TiO_2, **b** TiO_2, and **c** Ce-TiO_2, corresponding to the edges L_2 and L_3 of Ti element after the background subtraction. The ratio of the integration of both blue areas (L_3 for Ti^{3+} and L_2 for Ti^{4+}) provides information about the oxidation level of Ti cations. (Source data are provided as a Source Data file.)

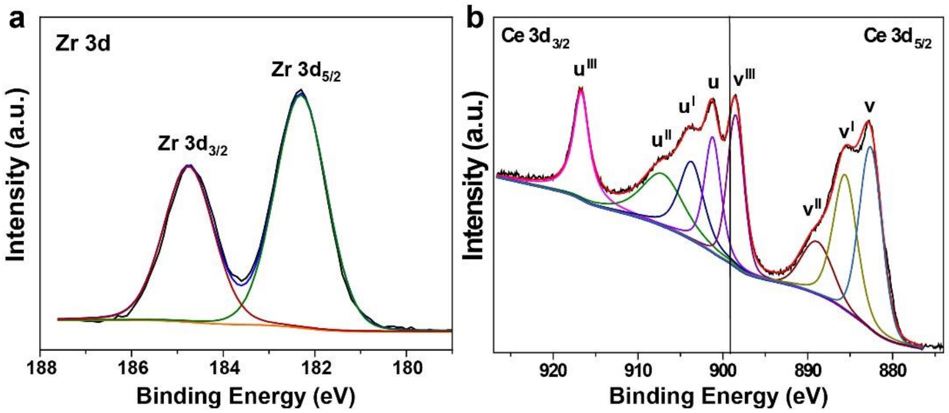

Supplementary Figure 7.XPS characterizations of the catalysts. a Zr 3d XPS spectra of Zr-TiO_2 sample. **b** Ce 3d XPS spectra of Ce-TiO_2 sample. (Source data are provided as a Source Data file.)

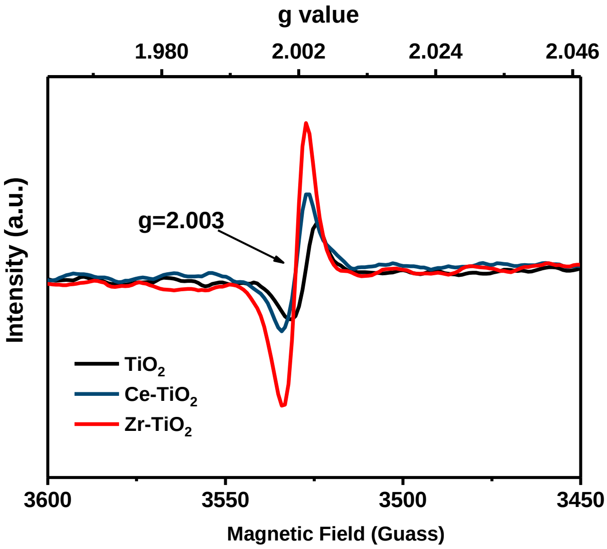

Supplementary Figure 8. Electron-paramagnetic resonance (EPR) spectra of the catalysts. EPR spectra of the undoped TiO_2, Zr-TiO_2 and Ce-TiO_2 samples. (Source data are provided as a Source Data file.)

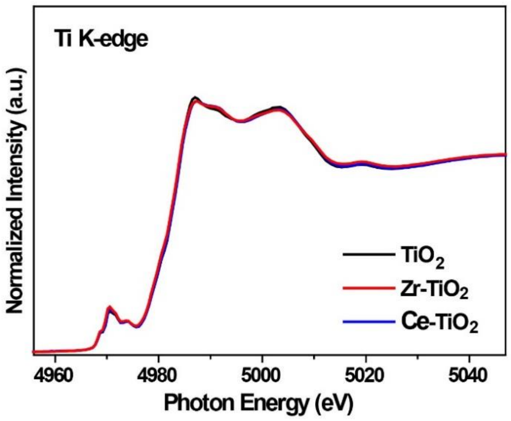

Supplementary Figure 9. The Ti K-edge XANES of the undoped TiO_2, Zr-TiO_2 and Ce-TiO_2 samples. (Source data are provided as a Source Data file.)

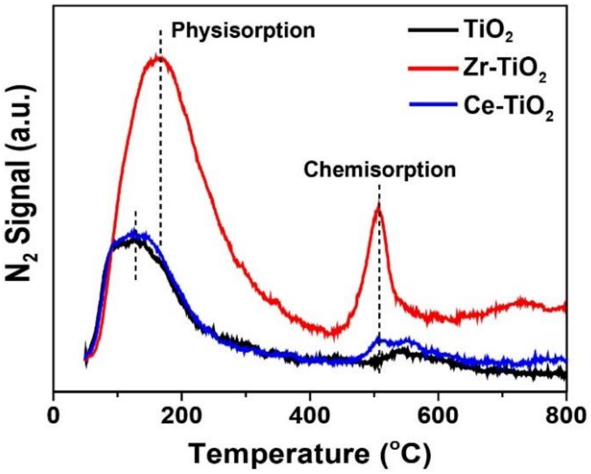

Supplementary Figure 10. The N_2-TPD signal of the undoped TiO_2, Zr-TiO_2 and Ce-TiO_2 samples. (Source data are provided as a Source Data file.)

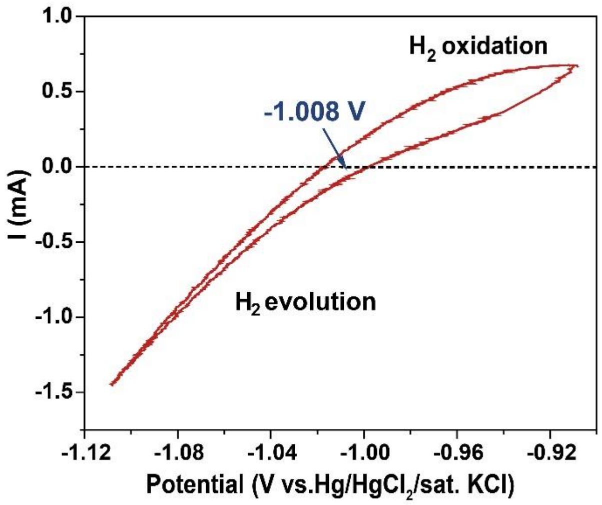

Supplementary Figure 11. Calibration of reference electrodes. The calibration of Hg/$HgCl_2$/saturated KCl reference electrode with respect to reversible hydrogen electrode (RHE). Thus, in 0.1 M KOH, $E_{(RHE)} = E_{SCE} + 1.008$ V. (Source data are provided as a Source Data file.)

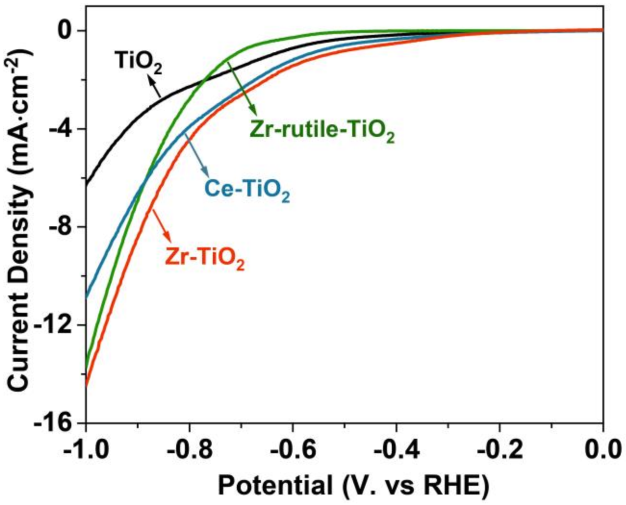

Supplementary Figure 12. Electrochemical tests of the catalysts. LSV curves of undoped TiO_2, Ce-TiO_2, Zr-rutile-TiO_2, and Zr-TiO_2. (Source data are provided as a Source Data file.)

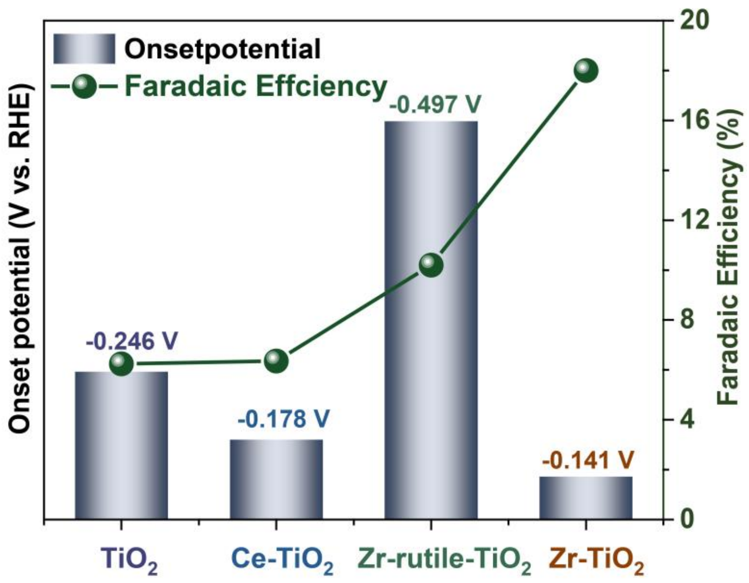

Supplementary Figure 13. Electrochemical N_2RR results of the catalysts. Onset potentials (at 50 μA·cm^{-2}) and corresponding faradaic efficiency values (green dots) with different catalysts at −0.45 V vs. RHE at room temperature and atmospheric pressure. (Source data are provided as a Source Data file.)

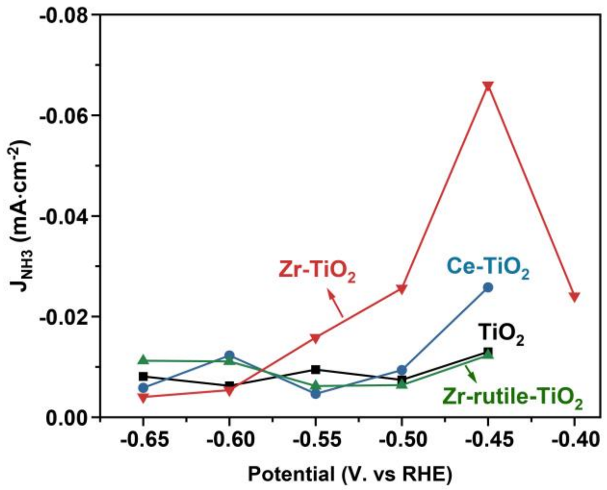

Supplementary Figure 14. NH_3 partial current density (J_{NH3}) of undoped TiO_2, Ce-TiO_2, Zr-rutile-TiO_2, and Zr-TiO_2 at different applied potentials. (Source data are provided as a Source Data file.)

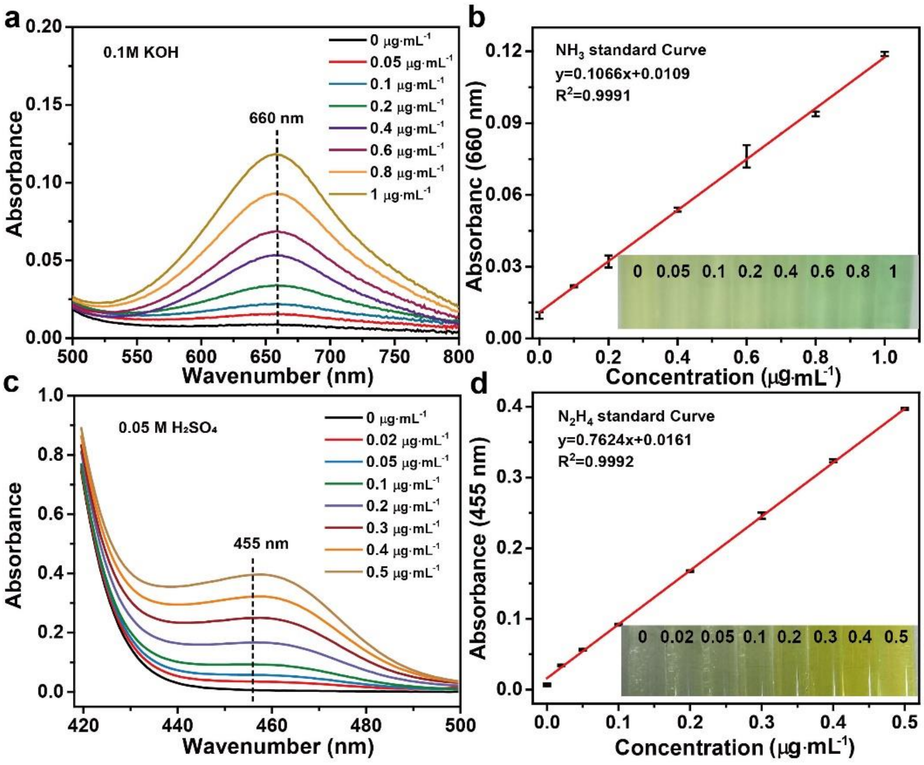

Supplementary Figure 15. Quantification of ammonia and hydrazine. a UV–Vis spectroscopy of colorimetry and **b** the calibration of colorimetric NH_3 assay by Salicylic acid spectrophotometry. **c** UV–Vis spectra curves, and **d** the calibrated N_2H_4 assay. (Source data are provided as a Source Data file.)

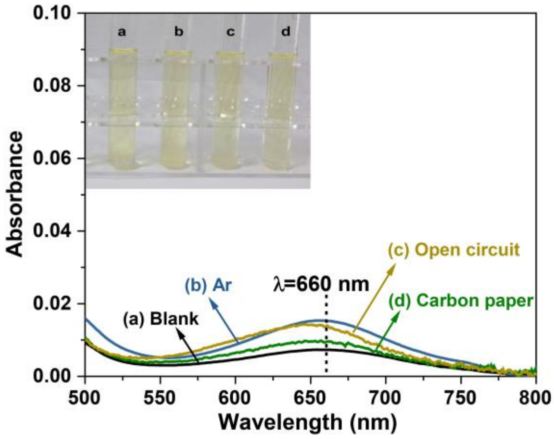

Supplementary Figure 16. Photograph and absorption spectra of the colorimetric assays: a without electrocatalytic treatment (Blank); **b** in an Ar-saturated electrolyte at –0.45 V vs. RHE for 3 h; **c** in a N_2-saturated electrolyte at open-circuit for 3 h; and **d** only the Nafion-dispersed carbon paper in a N_2-saturated electrolyte at –0.45 V vs. RHE for 3 h. A is Abbreviations for absorbance. The UV–Vis absorbance at 660 nm of these samples were recorded as: **a** 0.0076, **b** 0.0153, **c** 0.0143 and **d** 0.0093, respectively. (Source data are provided as a Source Data file.)

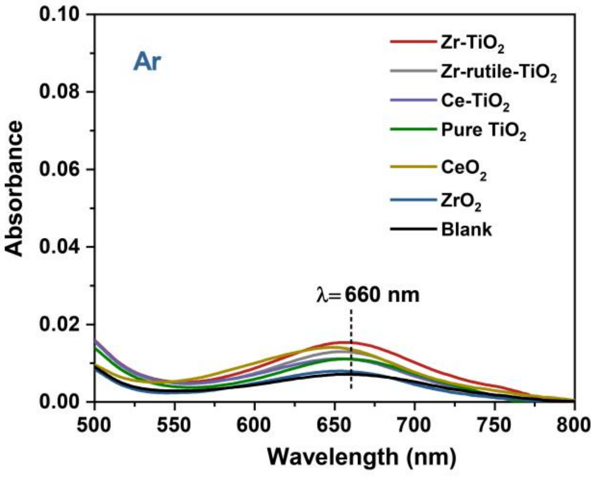

Supplementary Figure 17. NH_3 detection of the electrolyte from all samples under Ar control. UV–Vis absorption spectra of the KOH electrolyte stained with indophenol indicator after charging at –0.45 V vs. RHE for 3 h under Ar controls for ammonia determination. (Source data are provided as a Source Data file.)

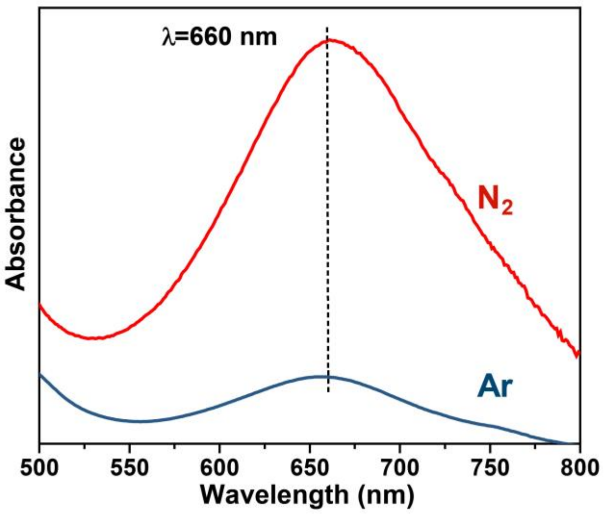

Supplementary Figure 18. NH_3 detection of the electrolyte from Zr-TiO_2 under Ar and N_2 control. UV–Vis absorption spectra of the electrolyte from the Zr-TiO_2 catalysts stained with indophenol indicator after charging at –0.45 V vs. RHE by using N_2 and Ar for ammonia determination. (Source data are provided as a Source Data file.)

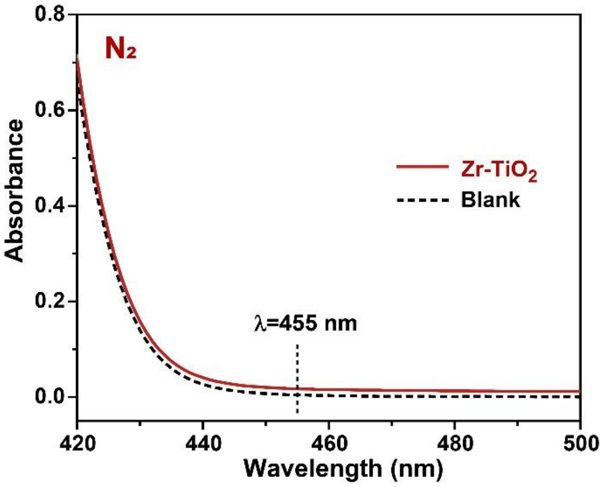

Supplementary Figure 19. N_2H_4 detection of the electrolyte from Zr-TiO_2. UV–Vis absorption spectra of the electrolyte for Zr-TiO_2 after N_2RR test at –0.45 V vs. RHE for hydrazine determination under N_2 controls. (Source data are provided as a Source Data file.)

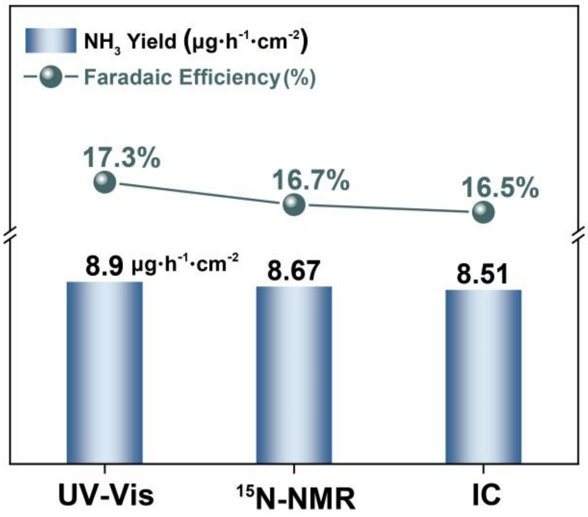

Supplementary Figure 20. Comparison of different ammonia detection methods. Comparison of the NH_3 yield rate (blue bars) and Faradaic efficiency (green dots) of Zr-TiO_2 calculated by utilizing different detection methods at –0.45 V vs. RHE. (Source data are provided as a Source Data file.)

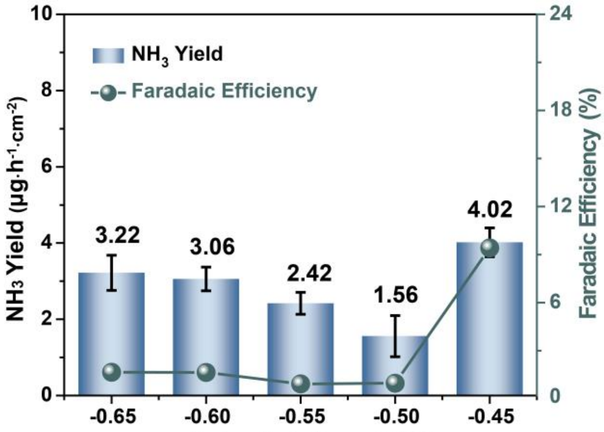

Supplementary Figure 21. N_2 fixation results of the Zr-rutile-TiO_2 catalyst. Yield of NH_3 (blue bars, left *y*-axis) and Faradaic efficiency (green dots, right *y*-axis) of Zr-rutile-TiO_2 at each given potentials. (Source data are provided as a Source Data file.)

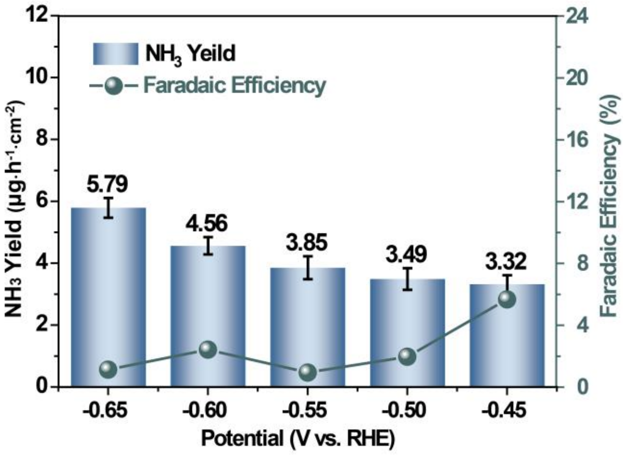

Supplementary Figure 22. **N_2 fixation results of the Ce-TiO_2 catalyst.** Yield of NH_3 (blue bars, left *y*-axis) and Faradaic efficiency (green dots, right y-axis) of Ce-TiO_2 at each given potentials. (Source data are provided as a Source Data file.)

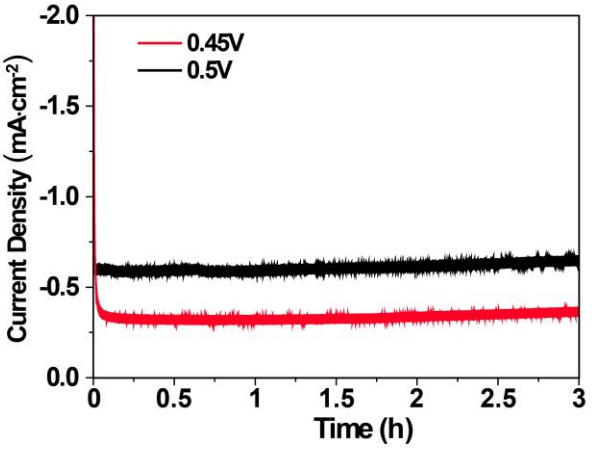

Supplementary Figure 23. Chrono-amperometry results at the corresponding potentials. (Source data are provided as a Source Data file.)

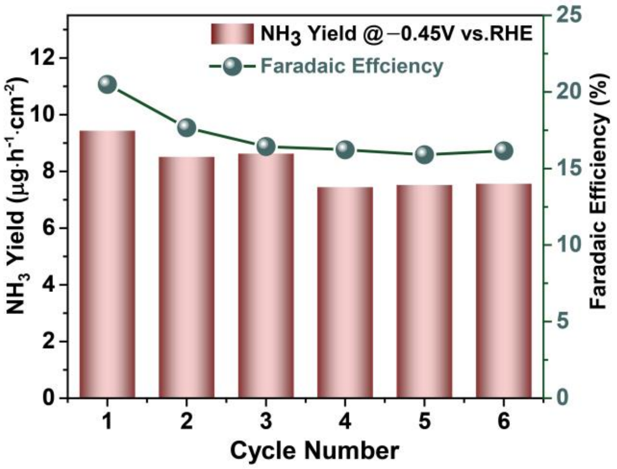

Supplementary Figure 24. The cycling stability results in N_2-saturated 0.1 M KOH (–0.45 V vs. RHE). (Source data are provided as a Source Data file.)

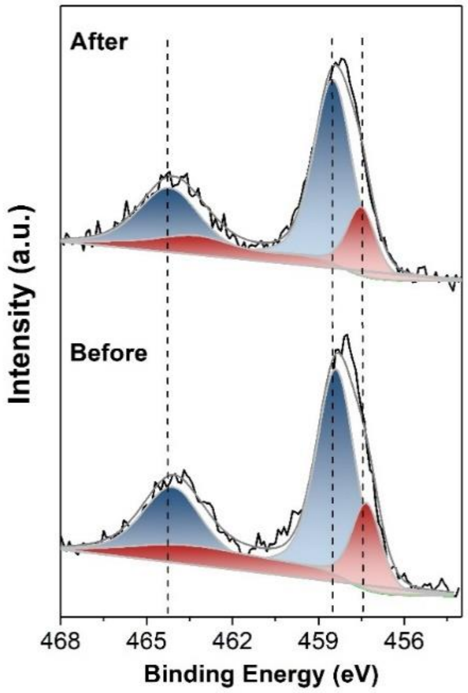

Supplementary Figure 25. Durability measurement. Ti 2p XPS spectrum of the Zr-TiO_2 catalysts before and after the electrolytic reaction. (Source data are provided as a Source Data file.)

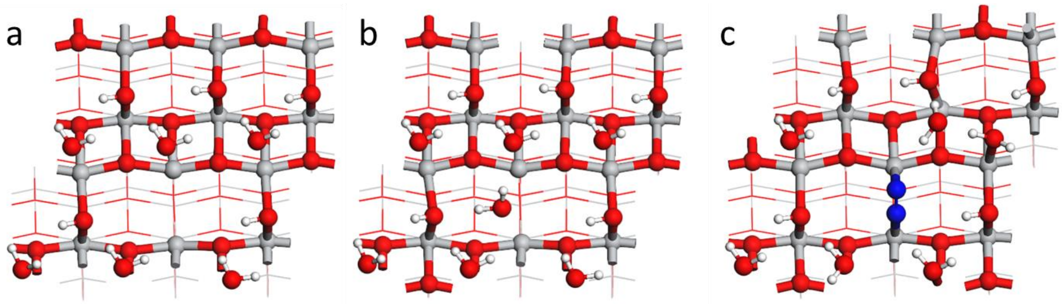

Supplementary Figure 26. The corresponding structures for the competitive adsorption of water and nitrogen on the A(101)-Vo. The competitive adsorption of water and nitrogen on the A(101)-Vo model, where the bridge O* is hydrogenated and the water adsorb on all the exposed titanium. **a** adsorption site; **b** water adsorption; **c** N_2 adsorption.

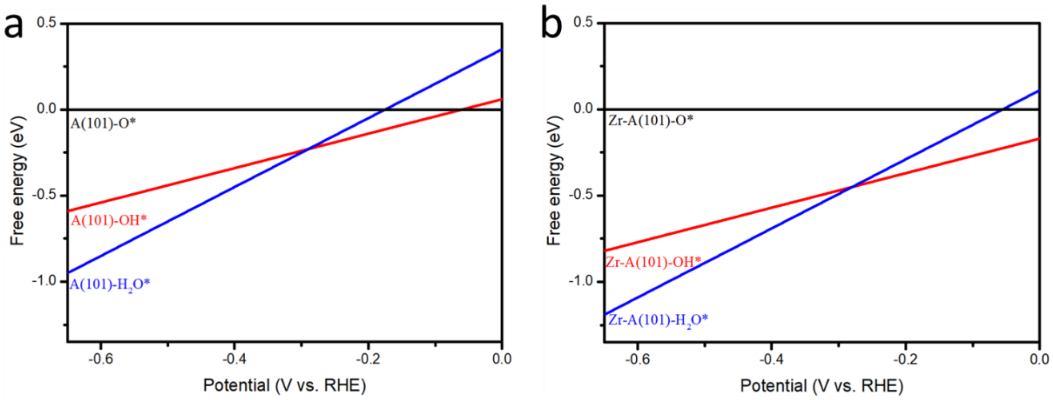

Supplementary Figure 27. Free energy diagrams of the pristine surface O* and hydrogenated surfaces OH* and H_2O*. Free energy diagrams of the OH* (i.e., the red line) and H_2O* (i.e., the blue line) for **a** A(101) surface and **b** Zr-A(101) surface under different potentials with respect to the corresponding pristine surface O* (i.e., the black line). (The strained A(101)-Vo in Supplementary Table S3 is used to present Zr-A(101)-Vo).

Supplementary Tables

Supplementary Table 1. The electronic binding energy of N_2, N_2H, NH_2, NH_3 on four candidates. All energies are with respect to the gas phase molecules of N_2 and H_2. (Unit: eV)

	A(101)-Vo (bi-Ti^{3+})	A(101)-Vo (single-Ti^{3+})	R(110)-Vo (bi-Ti^{3+})	R(110)-2Vo (bi-Ti^{3+})
$E_{ad}(N_2)$	–0.54	–0.52	–0.57	–0.69
$E_{ad}(N_2H)$	–0.46	–0.18	0.02	–0.60
$E_{ad}(NH_2)$	–2.53	–2.53	–2.38	–2.49
$E_{ad}(NH_3)$	–2.51	–2.51	–2.68	–2.42

Supplementary Table 2. The DFT optimized lattice constants of Zr-doped anatase TiO_2 with increasing numbers of doped Zr^{4+} atom number (as *x*). A 2 x 2 x 1 supercell model ($Zr_xTi_{16-x}O_{32}$) was used when the bulk lattice was optimized.

x	a = b (Å)	c (Å)
0	3.867	9.582
2	3.889	9.696
3	3.903	9.725
4	3.915	9.793

Supplementary Table 3. The influence of Zr^{4+} on the formation energy of surface oxygen vacancy. Oxygen vacancy formation energies on some representatives of the strained A(101) surfaces with the numbers of Zr^{4+} on the surface x and the numbers of Zr^{4+} on the subsurface y: (a) $x = 0$, $y = 0$; (b) $x = 2$, $y = 4$; (c) $x = 3$, $y = 4$; (d) $x = 4$, $y = 4$. The strained anatase (101) surfaces are cleaved from the bulk with lattice constants (a, b and c) 1.023[a] times larger than those of the DFT optimized structure with fully relaxed atomic positions.

	Geometry structure[b]	Oxygen vacancy formation energy[c] (eV)
a	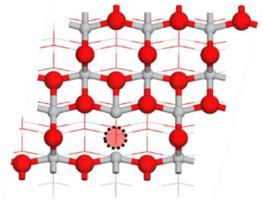	3.63
b	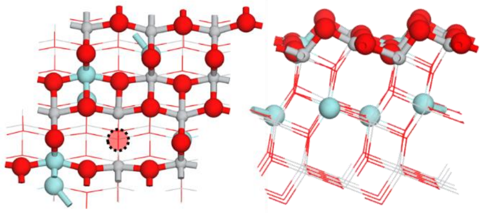	3.63
c	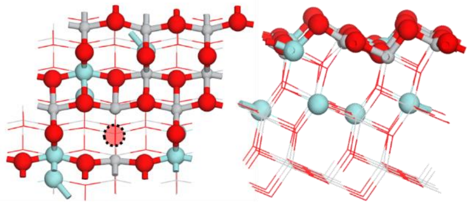	3.57
d	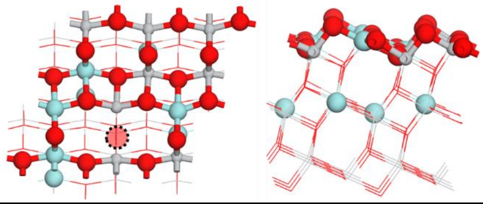	3.68

Notes:

[a] The expansion ratio of 1.023 corresponds to the XRD results that the corresponding interplanar spacing values of the (101) planes changed from 0.351 nm for undoped TiO_2 to 0.359 nm for Zr-TiO_2. It is assumed here that the lattice constants (a, b, c) expanded at the same ratio in all directions.

[b] The red, grey spheres represent oxygen, titanium atoms on the surface, respectively. The blue spheres represent the zirconium atoms on the surfaces or subsurfaces.

[c] The formation energy is presented as energy that a pure surface loses 0.5*O_2 to leave one oxygen vacancy on the surface (see eq. 4 and 5). The formation energy of oxygen vacancy at the optimized lattice constants is 3.90 eV. Hence expansion and Zr-doping lower the formation energy by 0.22–0.33 eV.

Supplementary Table 4. The competitive adsorptions of H_2O and H on the A(101)-Vo surface. The competitive adsorptions of H_2O and H on the A(101)-Vo surface.

**Reaction**[a]	**Δ*G*/eV**
$N_2(g) + H_2O^* = N_2^* + H_2O(l)$	0.04[b]
$(H^+ + e^-) + Vo = H^*$	0.19
$(H^+ + e^-) + H^* = H_2(g) + Vo$	0.04

[a] * refers to adsorbed species.

[b] For convenience, here we assume the chemical potential of the water in solution is equal to 3.169 kPa as pure liquid water at room temperature.[45] Note that, on the empty A(101)-Vo surface, we find that the binding of water is much stronger than N_2. However, under working conditions, the hydrogenation of bridge O* and the adsorption of water on the A(101)-Vo are quite easy. Thus, we further check the competitive adsorption of water and nitrogen on the A(101)-Vo model, where the bridge O* is hydrogenated and the water adsorb on all the exposed titanium, as shown in Supplementary Fig. 25a. The corresponding adsorption structure of water and nitrogen are as shown in Supplementary Fig. 25b and c.

Supplementary Table 5. Summary of the representative reports on electrochemical N_2 reduction in aqueous solution at low temperature and ambient pressure.

Temp.	Catalyst	[a]pH	Electrolyte	Yield	FE (%)	Potential	Ref.
**25 ºC**	**Zr-TiO_2**	**13**	**0.1 M KOH**	**~8.90 μg h^{-1} cm^{-2}**	**17.3**	**–0.45 V vs. RHE**	**This work**
25 ºC	Fe_2O_3-CNT	~12	0.5 M KOH	0.41 μg h^{-1} cm^{-2}	0.03	–1.0 Vvs. Ag/AgCl	Ref.[1]
30 ºC	Ru/Ti	0	0.5 M H_2SO_4	7.31 μg h^{-1} cm^{-2}	N/A	N/A	Ref. [2]
25 ºC	Fe_3O_4/Ti	~6.2	0.1 M Na_2SO_4	3.43 μg h^{-1} cm^{-2}	2.6	–0.4 V vs. RHE	Ref. [3]
25 ºC	MoS_2	~6.2	0.1 M Na_2SO_4	4.95 μg h^{-1} cm^{-2}	1.17	–0.5 V vs. RHE	Ref. [4]
25 ºC	$Pd_{0.2}Cu_{0.8}$/[c]rGO	13	0.1 M KOH	2.8 μg h^{-1} mg^{-1}	<1.5	–0.2 V vs. RHE	Ref. [5]
25 ºC	TiO_2-rGO	~6.2	0.1 M Na_2SO_4	15.1 μg h^{-1} mg^{-1}	3.3	–0.9 V vs. RHE	Ref. [6]
25 ºC	a-Au/CeO_x-[c]rGO	1	0.1 M HCl	8.3 μg h^{-1} mg^{-1}	10.1	–0.2 V vs. RHE	Ref. [7]
[d]RT	Pd/C	7.2	0.1 M [e]PBS	4.5 μg h^{-1} cm^{-2}	8.2	0.1 V vs. RHE	Ref. [8]
RT	[f]CNSs	N/A	0.25 M $LiClO_4$	97.2 μg h^{-1} cm^{-2}	11.6	–1.19 V vs. RHE	Ref. [9]
RT	[g]NCM-Au	0	1 M HCl	0.36 g m^{-2} h^{-1}	22	–0.2 V vs. RHE	Ref. [10]
RT	[h]Ru SAs/N−C	1	0.05 M H_2SO_4	30.8 μg h^{-1} cm^{-2}	29.6	–0.2 V vs. RHE	Ref. [11]
RT	Fe−N/C	13	0.1 M KOH	34.8 μg h^{-1} mg^{-1}	9.28	–0.2 V vs. RHE	Ref. [12]

Notes:
a: The pH values of the electrolytes in different literatures were calculated based on the corresponding electrolytes.
b: PEBCD, poly (N–ethyl–benzene–1, 2, 4, 5–tetracarboxylic diimide).
c: rGO, reduced graphite oxide.
d: Room temperature.
e: Phosphate buffer solution
f: Nitrogen-doped nanoporous carbon membranes.
g: N-doped carbon nanospikes.
h: Ru single atoms on nitrogen-doped carbon.

Supplementary Notes

Supplementary Note 1 Calculation of the standard potential of N_2 reduction reaction:

The "standard potential" value of 0.056 V vs. RHE can be calculated in our solution as follows:

N_2 (g) + 6 H_2O (l) + 6 e^- → 2 NH_3 (g) + 6 OH^- (aq) $\Delta G^o = 446.6$ kJ mol^{-1}

$E^o = -\Delta G^o/n\mathrm{F} = -0.771$ V,   versus Standard hydrogen electrode (vs. SHE)

$E = E^o - \mathrm{RT}/n\mathrm{F} \times \ln[c_{(OH-)}]^6 + 0.059\ \mathrm{V} \times \mathrm{pH} = 0.056$ V,   versus Reversible hydrogen electrode (vs. RHE)

Note: The standard Gibbs energy of formation at 298.15 K for these compound and aqueous ions were based on *CRC Handbook of Chemistry and Physics*, the 85th Edition[13].

ΔG^o (N_2) = 0 kJ mol^{-1}, ΔG^o (H_2O) = –237.1 kJ mol^{-1}, ΔG^o (NH_3) = –16.4 kJ mol^{-1}, ΔG^o (OH^-) = –157.2 kJ mol^{-1}

And, n is the number of transferred electrons (n = 6), F is the Faraday constant (96485 C mol^{-1}), R is the molar gas constant (8.314 J mol^{-1} K^{-1}), $c_{(OH-)} = 10^{-1}$ M and the pH is 13. Thus, the corresponding thermodynamic equilibrium potentials is calculated to be 0.056 V vs. RHE.

Supplementary Note 2 Calculation of the total ammonia production in 3 h of electrochemical test:

As the volume of electrolyte was 30 mL, the amount of NH_3 produced in our experiment was calculated as follows.

$m_{\text{NH3 (yield)}} = \Delta c_{\text{NH3 (yield)}} \times V_{\text{aq}} = (c_{\text{NH3 (yield, N2)}} - c_{\text{NH3 (yield, Ar)}}) \times 30 \text{ mL} = 9.156\ \mu\text{g}$

$n_{\text{NH3 (yield)}} = m_{\text{NH3}} / M_{\text{NH3}} = 9.156\ \mu\text{g} / (17.031 \text{ g / mol}) = 0.538\ \mu\text{mol}$

Supplementary References

1. Chen, S. et al. Room-temperature electrocatalytic synthesis of NH_3 from H_2O and N_2 in a gas–liquid–solid three-phase reactor. *ACS Sustain. Chem. Eng.* **5**, 7393-7400 (2017).
2. Kugler, K., Luhn, M., Schramm, J.A., Rahimi, K. & Wessling, M. Galvanic deposition of Rh and Ru on randomly structured Ti felts for the electrochemical NH_3 synthesis. *Phys. Chem. Chem. Phys.* **17**, 3768-3782 (2015).
3. Liu, Q. et al. Ambient N_2 fixation to NH_3 electrocatalyzed by a spinel Fe_3O_4 nanorod. *Nanoscale* **10**, 14386-14389 (2018).
4. Zhang, L. et al. Electrochemical ammonia synthesis via nitrogen reduction reaction on a MoS_2 catalyst: Theoretical and experimental studies. *Adv. Mater.* **30**, e1800191 (2018).
5. Shi, M.-M. et al. Anchoring PdCu amorphous nanocluster on graphene for electrochemical reduction of N_2 to NH_3 under ambient conditions in aqueous solution. *Adv. Energy Mater.* **8**, 1800124 (2018).
6. Zhang, X. et al. TiO_2 nanoparticles–reduced graphene oxide hybrid: An efficient and durable electrocatalyst toward artificial N_2 fixation to NH_3 under ambient conditions. *J. Mater. Chem. A* **6**, 17303-17306 (2018).
7. Li, S. J. et al. Amorphizing of Au nanoparticles by CeO_x-RGO hybrid support towards highly efficient electrocatalyst for N_2 reduction under ambient conditions. *Adv. Mater.* **29**, 1700001 (2017).
8. Wang, J. et al. Ambient ammonia synthesis via palladium-catalyzed electrohydrogenation of dinitrogen at low overpotential. *Nat. Commun.* **9**, 1795 (2018).
9. Song, Y. et al. A physical catalyst for the electrolysis of nitrogen to ammonia. *Sci. Adv.* **4**, e1700336 (2018).
10. Wang, H. et al. Ambient electrosynthesis of ammonia: Electrode porosity and composition engineering. *Angew. Chem. Int. Ed.* **57**, 12360-12364 (2018).
11. Geng, Z. et al. Achieving a record-high yield rate of 120.9 μg_{NH3} $mg_{cat.}^{-1}$ h^{-1} for N_2 electrochemical reduction over Ru single-atom catalysts. *Adv. Mater.* e1803498 (2018).
12. Wang, Y. et al. Rational design of Fe–N/C hybrid for enhanced nitrogen reduction electrocatalysis under ambient conditions in aqueous solution. *ACS Catal.* **9**, 336-344 (2018).
13. Lide, D. R. et al. *CRC Handbook of Chemistry and Physics*, (85th ed.). CRC Press. pp. 6–8. ISBN 978-0-8493-0485-9 (2004).
